# Mitochondria Targeting with Luminescent Rhenium(I) Complexes

**DOI:** 10.3390/molecules22050809

**Published:** 2017-05-15

**Authors:** Joanna Skiba, Tytus Bernaś, Damian Trzybiński, Krzysztof Woźniak, Giarita Ferraro, Daniela Marasco, Antonello Merlino, Marsel Z. Shafikov, Rafał Czerwieniec, Konrad Kowalski

**Affiliations:** 1 Faculty of Chemistry, Department of Organic Chemistry, University of Łódź, Tamka 12, 91-403 Łódź, Poland; asiaskiba@02.pl; 2 Nencki Institute of Experimental Biology, Polish Academy of Sciences, ul. Pasteura 3, 02-093 Warszawa, Poland; t.bernas@nencki.gov.pl; 3 Faculty of Chemistry, Biological and Chemical Research Centre, University of Warsaw, Żwirki i Wigury 101, 02-089 Warszawa, Poland; trzybinski@chem.uw.edu.pl (D.T.); kwozniak@chem.uw.edu.pl (K.W.); 4 Department of Chemical Sciences, University of Naples Federico II, Complesso Univ. di Monte Sant’ Angelo, Via Cintia, I-80126 Napoli, Italy; giarita.ferraro@unina.it (G.F.); antonello.merlino@unina.it (A.M.); 5 Department of Pharmacy, University of Naples Federico II, Via Mezzocannone 16, 80134 Napoli, Italy; daniela.marasco@unina.it; 6 CIRPEB: Centro Interuniversitario di Ricerca sui Peptidi Bioattivi, Via Mezzocannone 16, I-80134 Napoli, Italy; 7 CNR Institute of Biostructures and Bioimages, Via Mezzocannone 16, I-80134 Napoli, Italy; 8 Department of Technology of Organic Synthesis, Institute of Chemical Engineering, Ural Federal University, 19 Mira Str., 620002 Ekaterinburg, Russia; shafikoff@gmail.com; 9 Institut für Physikalische und Theoretische Chemie, Universität Regensburg, Universitätsstraße 31, D-93040 Regensburg, Germany; 10 Lehrstuhl für Anorganische Chemie I, University of Bayreuth, D-95440 Bayreuth, Germany

**Keywords:** bioorganometallics, rhenium complexes, ferrocene, confocal microscopy, luminescence, mitochondria staining, phototoxicity

## Abstract

Two new neutral *fac*-[Re(CO)_3_(phen)L] compounds (**1**,**2**), with phen = 1,10-phenanthroline and L = O_2_C(CH_2_)_5_CH_3_ or O_2_C(CH_2_)_4_C≡CH, were synthetized in one-pot procedures from *fac*-[Re(CO)_3_(phen)Cl] and the corresponding carboxylic acids, and were fully characterized by IR and UV-Vis absorption spectroscopy, ^1^H- and ^13^C-NMR, mass spectrometry and X-ray crystallography. The compounds, which display orange luminescence, were used as probes for living cancer HeLa cell staining. Confocal microscopy revealed accumulation of both dyes in mitochondria. To investigate the mechanism of mitochondrial staining, a new non-emissive compound, *fac*-[Re(CO)_3_(phen)L], with L = O_2_C(CH_2_)_3_((C_5_H_5_)Fe(C_5_H_4_), i.e., containing a ferrocenyl moiety, was synthetized and characterized (**3**). **3** shows the same mitochondrial accumulation pattern as **1** and **2**. Emission of **3** can only be possible when ferrocene-containing ligand dissociates from the metal center to produce a species containing the luminescent *fac*­[Re(CO)_3_(phen)]^+^ core. The release of ligands from the Re center was verified in vitro through the conjugation with model proteins. These findings suggest that the mitochondria accumulation of compounds **1**–**3** is due to the formation of luminescent *fac*-[Re(CO)_3_(phen)]^+^ products, which react with cellular matrix molecules giving secondary products and are uptaken into the negatively charged mitochondrial membranes. Thus, reported compounds feature a rare dissociation-driven mechanism of action with great potential for biological applications.

## 1. Introduction

**Figure 1:**
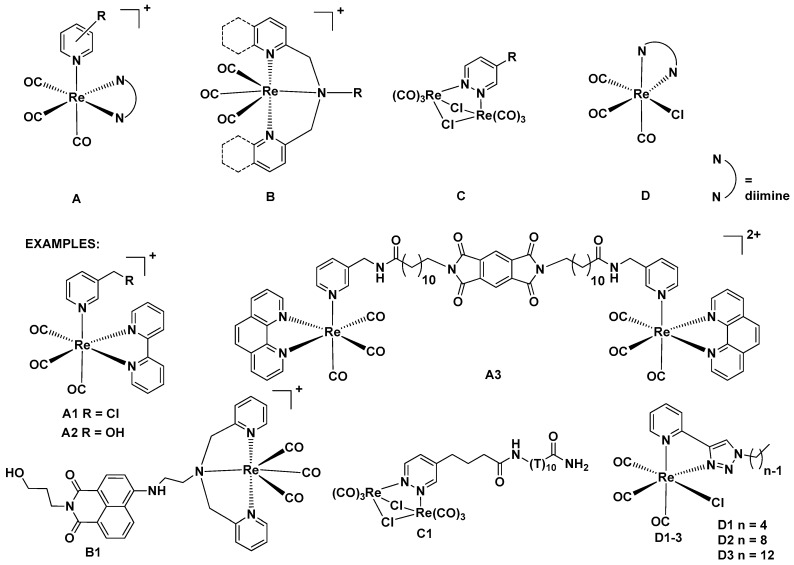
Basic structures of luminescent *fac*-{Re(CO)_3_} complexes for cell bioimaging and their examples. Four basic classes of luminescent bioprobes with fac-{Re(CO)3} cores (**A**–**D**).

Luminescent transition metal complexes have been extensively used in organic light-emitting diodes (OLEDs) [1], as optical sensors [2], and in solar cell materials [3]. Over the last decade, this class of materials has attracted an increased attention as luminescent [4,5,6,7,8,9,10,11,12,13] and vibrational-luminescent [14] probes for bioimaging. In particular, *fac*-[Re(CO)_3_(N˄N)L] (N˄N = aromatic diimine and L = ancillary ligand) complexes have been investigated for bioimaging applications [4,13]. Upon photoexcitation, *fac*-[Re(CO)_3_(N˄N)L] complexes display green to orange phosphorescence stemming from triplet metal-to-ligand charge-transfer (^3^MLCT) states [15,16,17,18]. The emission occurs at much longer wavelengths and with distinctly longer decay times than the cell autofluorescence. Both effects are beneficial for bioimaging applications [5]. Studies on luminescent rhenium complexes have also had a great impact on development of ^99m^Tc and ^186,188^Re containing radiopharmaceuticals [13,19]. A literature survey shows that four basic classes of luminescent bioprobes with *fac*-{Re(CO)_3_} cores can be identified (Figure 1A–D).

Most of the research has been focused on cationic complexes (classes **A** and **B** in Figure 1) which preferably stain mitochondria [20,21,22,23]. Relevant examples of such compounds comprise cationic **A1** [20] and **A2** [21] species which stain mitochondria of MCF-7 cells, and dication **A3**, which accumulates in endoplasmatic retictulum (ER), Golgi apparatus and mitochondria [22]. Staining mechanisms significantly depend on many factors, e.g. probe concentration or ligand reactivity [20,23,24]. For example, it has been found that compound **A1** covalently binds to thiol-rich mitochondrial components that allow for efficient accumulation [20]. A remarkable example of class **B** compounds is the naphthalimide derivative **B1** which shows a concentration-dependent pathway of accumulation in cellular compartments. At low concentrations, **B1** stains smooth endoplasmic membranes of osteoarthritic cells, while at higher concentrations it rapidly binds to mitochondria membranes [23]. Neutral complexes (classes **C** and **D** in Figure 1) have been found to accumulate in nucleus and perinuclear compartments of the cell [24,25]. Dirhenium piridazine complex **C1** stain the cytoplasm and nucleus [25], and compounds **D1**–**3** stain the perinuclear region of MDA-MB-231 cells [24]. It has been observed that the cellular uptake of **D1**–**3** compounds increases as the alkyl chain length of the ligand increases [24]. Moreover, for several compounds photocytotoxic and/or cytotoxic activity has been observed [26,27,28,29,30,31,32,33,34,35,36,37].

Herein we report the synthesis and characterization, including X-ray structure analysis, of three new *fac*-[Re(CO)_3_(1,10-phenantroline)L] complexes, hereafter called **1**–**3**, with different ancillary ligands: L = heptanoic acid (**1**), hept-6-ynoic acid (**2**), and 4-(1-ferrocenyl)-butanoic acid (**3**), respectively. Complexes **1** and **2** are luminescent, whereas complex **3**, comprising of a quenching ferrocenyl moiety, does not [38]. Accumulation of **1**–**3** in mitochondria is observed for HeLa cells via confocal microscopy. Upon prolonged laser irradiation, **2** displays photocytotoxic activity. Comparative analysis of the data collected for the three compounds and results obtained for the adducts of **1** and **3** with model proteins enables us to propose a mechanism explaining the molecular basis of the mitochondrial accumulation of **1**–**3**. 

## 2. Results and Discussion

### 2.1. Synthesis of Complexes ***1***–***3***

**Scheme 1:**
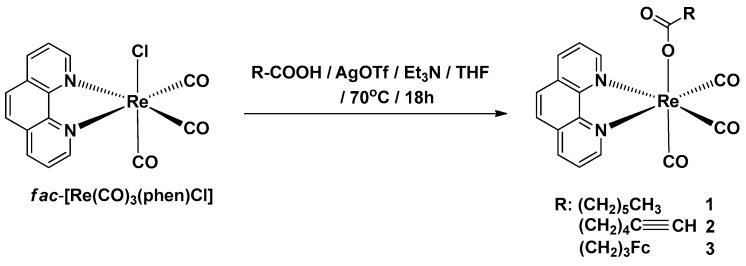
Synthesis of complexes **1**–**3**.

A literature survey shows that *fac*-[Re(CO)_3_(phen)L], where phen = 1,10-phenanthroline and L = carboxylate ligand, complexes can be prepared by: (i) reaction of *fac*-[Re(CO)_3_(phen)X] (with X = Cl or Br) precursor with a silver salt of respective carboxylic acid or *fac*-[Re(CO)_3_(phen)CF_3_SO_3_] precursor with carboxylic acid [39,40,41]; (ii) reaction of *fac*-[Re(CO)_3_(phen)(OH)] complex with ketene or lactide [42]; and (iii) reaction of an aqua [Re(OH_2_)_3_(CO)_3_]^+^ complex with a carboxylate-phenantroline ligand [43]. Our approach toward the synthesis of complexes **1**, **2**, and **3** was carried out according to Scheme 1.

We found that the *fac*-[Re(CO)_3_(phen)Cl] complex [44] readily reacts with carboxylic acids in THF in the presence of trimethylamine and silver trifluoromethanesulfonate at 70 °C to afford products **1**, **2** and **3** in a 95%, 65%, and 48% yield, respectively. The mechanism most probably involves a reaction of the carboxylic anion L^−^ and *fac*-[Re(CO)_3_(phen)]^+^ cation, generated in situ***,*** yielding the target complex. This approach represents a simple one-pot procedure for obtaining carboxylato-Re(CO)_3_(N-N) complexes. Compounds **1** and **2** are yellow air-stable solids, whereas complex **3** is an orange air-stable solid. The products were characterized by ^1^H-NMR, ^13^C-NMR, IR and UV-Vis absorption spectroscopy, mass spectrometry (MS), and elemental analysis. All analytical data confirmed the proposed structures. The IR spectra of complexes **1**–**3** in potassium bromide display three very strong ν_(C≡O)_ bands at 2027–1866 cm^−1^, strong ν_(C=O)_ bands at 1622–1606 cm^−1^ and solely in the case of alkyne complex **2**, a weak ν_(C≡C)_ band at 2119 cm^−1^. The ^1^H-NMR spectra of the three products are presented in Figures S1–S3 (SI). In the ^1^H-NMR spectrum of complex **2**, a characteristic alkyne signal at δ = 2.56 ppm is observed.

### 2.2. Single-Crystal X-ray Structural Analysis of Complexes ***1***–***3***

**Figure 2:**
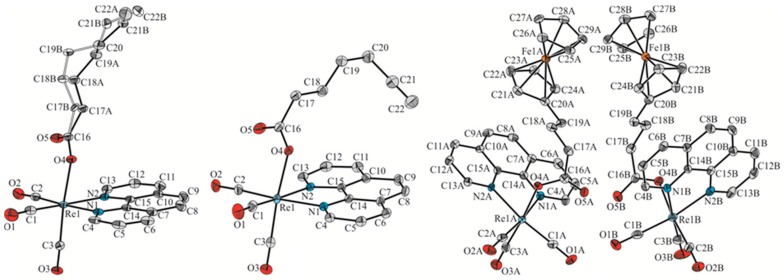
Oak Ridge thermal ellipsoid plot (ORTEP) diagram of **1** (**left**), **2** (**middle**) and **3** (**right**). Hydrogen atoms omitted for clarity. Displacement ellipsoids drawn at a 50% probability level.

The molecular structures of **1**–**3** were determined by single-crystal X-ray analysis. Crystals of **1** and **3** that were suitable for X-ray diffraction analysis were obtained from dimethyl sulfoxide-water mixture, whereas crystals of **2** were obtained by slow diffusion of *n*-pentane into a dichloromethane solution of **2**. It turned out that the investigated compounds crystallize in the P2_1_/n (**1** and **2**) or P2_1_ (**3**) space group of the monoclinic crystal system with one (**1** and **2**) or two molecules (**3**) of the given compound in the asymmetric unit, respectively. The Oak Ridge thermal ellipsoid plot (ORTEP) drawing of **1**, **2**, and **3** is shown with atom numbering in Figure 2. Details of crystallographic data and refinement parameters are summarized in Table S1. Geometrical parameters of **1**–**3** are presented in Table 1 and in Tables S2–S10 of Supplementary Materials.

All analyzed complexes exhibit typical distorted octahedral geometry with a *fac*-{Re(CO)_3_} arrangement and the axially oriented carboxylic ligand [42,45]. The hydrocarbon chain of the ligand in **1** is disordered over two positions with the site occupancy factors of 0.679(4) and 0.321(4). The whole ligand is strongly deflected relative to the phenantroline unit (Figure 2 (left)). In the case of **2** and **3**, the carboxylic ligands are directed towards the plane of the phenantroline ring system (Figure 2 (middle) and (right)). The above-mentioned differences in the spatial arrangement result from the packing effects of individual molecules in the crystal lattice. The length of the axial Re–O bond in **1** and **2** is 2.143(1) and 2.146(2) Å respectively, and is comparable to the value that is observed in similar rhenium complexes [42,45]. In the case of **3**, the Re–O bond length is slightly shorter and its value is 2.125(2) and 2.129(2) Å for the two molecules in the asymmetric unit (molecule A and B hereafter), respectively. X-ray diffraction confirmed the *O*-coordination mode of hept-6-ynoic acid ligand in **2**. The C(21)-C(22) terminal triple bond length is 1.174(6) Å. The iron atom in ferrocenyl groups of **3** is symmetrically displaced between the cyclopentadienyl ligands. For example, in molecule **3**A the Fe-Mp distances (Mp corresponds to the mid-point of the respective cyclopentadienyl ring) are 1.654(2) and 1.651(2) Å for the substituted and unsubstituted cyclopentadienyl ring, respectively. Similar results are found in molecule B. Ferrocenyl groups present in the asymmetric unit of **3** deviate from the eclipsed conformation by ϕ = 6° and 10° for the molecules A and B, respectively.

### 2.3. Photophysical Characterization

**Figure 3:**
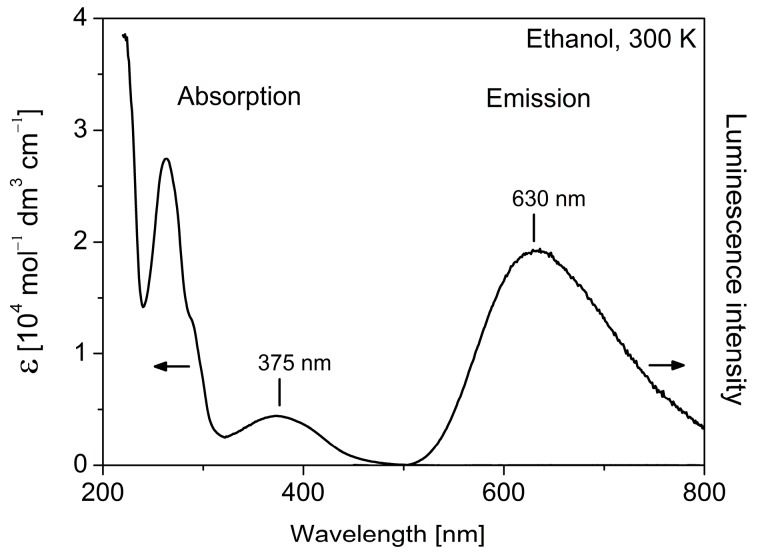
Absorption and luminescence spectra of **1** in ethanol at ambient temperature.

Electronic absorption spectra of complexes **1**–**3** are dominated by the features of the *fac*­[Re(CO)_3_(phen)] core. In particular, as it is shown in Figure 3 and Table 2, an intense ligand centered (^1^LC) π-π* transition of the phenanthroline ligand, with a maximum at 262 nm, is observed. The low-energy part of the spectra is dominated by a moderately strong broad metal-to-ligand charge-transfer (^1^MLCT) d(Re)-π*(phen) electronic transition centered at 375 nm [44,46]. The relatively very weak metal-centered d-d absorption of the ferrocenyl entity of **3**, with the molar absorption coefficient ε in the order of 10^2^ M^−1^·cm^−1^ [47], is obscured by the MLCT band. Due to the presence of the ferrocenyl moiety, compound **3** is not luminescent [38]. It becomes emissive after ferrocenyl ligand dissociation.

Complexes **1** and **2** display orange luminescence characteristics for the *fac*­[Re(CO)_3_(phen)] core [15,44]. The emission spectra measured in ethanol at ambient temperatures are broad with the maxima at 630 nm (Figure 3). The luminescence decay times τ found for both compounds amount to 60 ns. The relatively short values of τ are related to efficient vibrational quenching [1] of the emissive ^3^MLCT excited states of these molecules and correspond to low luminescence quantum yields of about 1%. Importantly, the obtained decay times measured for air-saturated solutions are almost equal to the τ values determined for degassed samples. This indicates a negligible influence of the molecular oxygen on emission quenching and enables us to use compounds **1** and **2** for confocal microscopy studies. 

**Figure 4:**
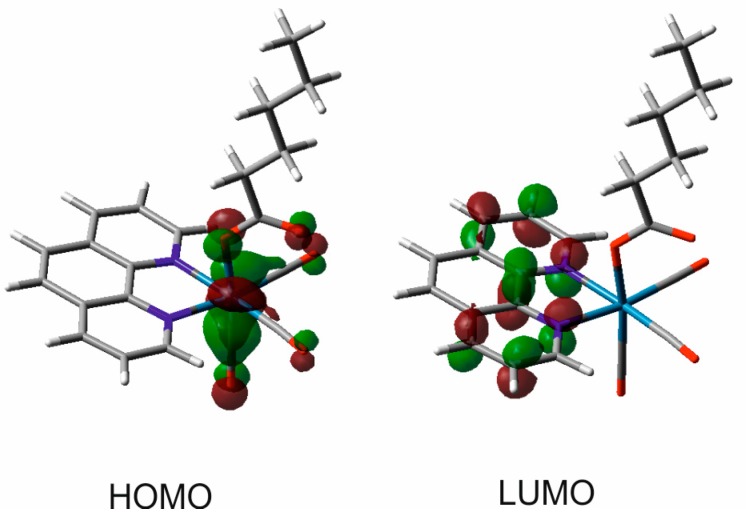
Frontier orbitals of **1** resulting from density-functional (DFT) calculations.

For completeness, it is mentioned that the MLCT character of the lowest excited states is also predicted by density-functional (DFT) calculations performed for **1** (Figure 4). Thus, HOMO of **1** is composed mainly from a rhenium 5d orbital perturbed with contributions from the CO and axial L ligands. LUMO represents a π* orbital of the phen ligand.

### 2.4. Confocal Microscopy Studies

**Figure 5:**
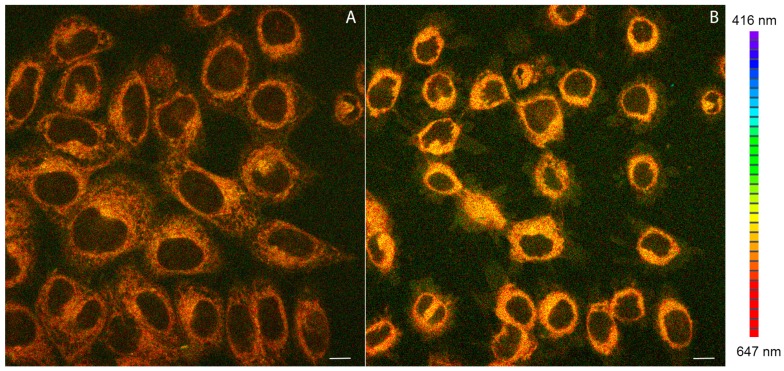
(**A**) Confocal luminescence image of living HeLa cells stained with **2**; (**B**) View of the photocytotoxic effect of **2** on HeLa cells after prolonged laser irradiation at 405 nm. Emission wavelengths are represented using a false color scale (right), with colors corresponding to spectral bands (416–647 nm total range). Scale bar 10 µm.

Complexes **1**–**3** were tested as luminescent probes for in vitro confocal microscopy bioimaging in HeLa cancer cells. All compounds under study were found in the interiors of the cells following 15 min incubation in a medium containing 200 nM of a given compound. Emission spectra recorded for the incubated cells were compatible with the luminescence spectra of **1** and **2** recorded in solution (Figure 5). In Figure 5A an exemplary confocal image of HeLa cells stained with **2** is reproduced. Analogous confocal images taken for compound **1** are shown in Figure S4A in Supplementary Materials.

**Figure 6:**
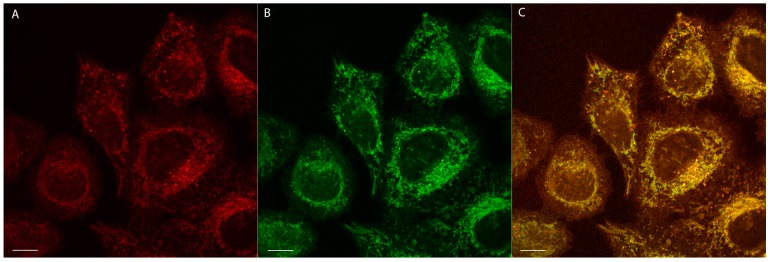
Mitochondria-targeting properties of **2**. (**A**) Co-localization images of HeLa cells stained with **2** (red) and (**B**) mitochondria-specific MitoTracker Green^®^ (green); (**C**) Merged image of (**A**) and (**B**). Scale bar—10 µm.

The luminescent probe is dominantly accumulated in mitochondria, which is confirmed by the characteristic granular staining pattern. In addition, diffuse (weak) emission stems also from cytoplasm and plasma membranes. The mitochondrial accumulation is further confirmed by a co-localization experiment using **2** and a mitochondria-specific fluorescent probe MitoTracker Green^®^ (Figure 6).

The co-localization of **2** and MitoTracker Green^®^ was quantified in HeLa cells by using the Manders (MCC) and Pearson correlation coefficient (PCC) [48]. The respective correlation coefficients were 0.83 ± 0.11 (MCC) and 0.73 ± 0.08 (PCC), both confirming the dominant localization of **2** in the mitochondria of HeLa cells. As expected, the staining pattern of compound **1** is the same as found for compound **2**. However, accumulation of neutral complexes in mitochondria is rather surprising, since the negative mitochondrial potential favors an uptake of positively charged species [20,21,22,23]. These considerations let us speculate that the mechanism of action for **1** and **2** involves a complex dissociation in the cell interior to form positively charged species having affinity to the mitochondrial membrane. This might be related to lability of the ancillary carboxylate ligands L. To verify this hypothesis, the non-emissive complex **3**, comprising a ferrocenyl substituted carboxylate ligand, can be used. For this compound, a similar granular staining pattern was observed (Figure S4B). This implies that the Re complex undergoes ligand dissociation yielding a phosphorescent cation comprising the *fac*-[Re(CO)_3_(phen)^+^] core. Probably, complexes **1** and **2** show the same mechanism of mitochondria staining.

To examine the role of the mitochondrial electric potential, living HeLa cells were preincubated with the mitochondrial uncoupler carbonyl cyanide-*p*-trifluoromethoxyphenylhydrazone (FCCP) [49], prior to staining with the luminophore **2**. FCCP acts as a proton transporter in mitochondria, dissipating the proton gradient across the mitochondrial membrane [49]. Depolarization of mitochondria treated with FCCP resulted in ca. 30% loss of the compound **2** luminescence intensity, compared to the control. However, the cellular localization of **2** in the presence and in the absence of FCCP remains the same (Figure S5). Such a behavior can only be rationalized by a diffusion of the positively charged Re-species from the depolarized mitochondria out to the surrounding cellular compartments. Nevertheless, a large amount of the compound accumulated in mitochondria is not responsive to the dissipation of mitochondrial potential. It can be surmised that they are bound to mitochondria by non-electrostatic forces. Accumulation of **2** did not induce loss of HeLa cell viability (Figure 5A). However, prolonged laser irradiation (405 nm, 150 µW, 3.75 J total dose) results in cell shrinkage and plasma membrane blebbing (Figure 5B).

**Figure 7:**
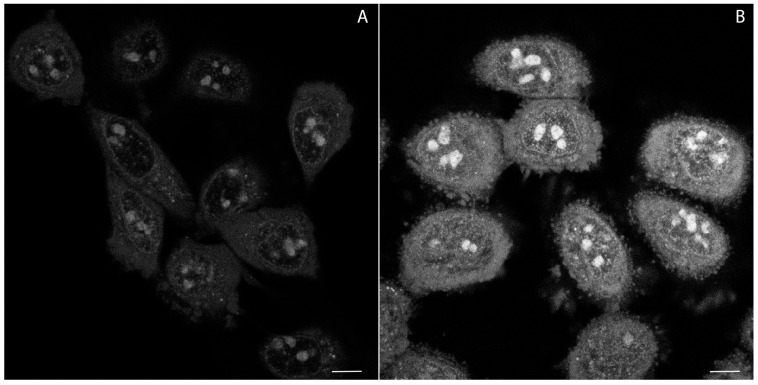
Production of reactive oxygen species (ROS) monitored with accumulation of ethidium (product of dihydroethidium (DHE) oxidation) after laser irradiation in the absence (**A**) and the presence (**B**) of compound **2**, in HeLa cells. Emission wavelengths are represented using a false colour. Scale bar—10 µm.

Thus, loss of plasma membrane integrity in the presence of **2** was investigated by using the propidium iodide (PI) exclusion test and wide-field image cytometry [50]. Accordingly, following illumination with 340–380 nm (35 µW) light for 360 s (12.6 J total dose), 14 ± 5% of HeLa cells lost their viability within 2 h, as compared to 5 ± 3% in the non-illuminated control. These results demonstrate that impairment of cell viability by accumulation of **2** is potentiated by phototoxic effects. This notion is compatible with production by **2** of superoxide radicals and other reactive oxygen species (ROS), detected with a dihydroethidium (DHE) staining assay after laser irradiation (Figure 7). This assay relies on DHE oxidation by cellular ROS. Oxidized DHE molecules become red fluorescent upon DNA and double stranded RNA intercalation, and as such can be detected with confocal microscopy.

Accordingly, Figure 7B shows about 2.04 ± 0.19 times more intense fluorescence in treated cells in comparison to the control population (Figure 7A). Confocal microscopy studies revealed some properties of assayed molecules in living cells. In order to investigate these proprieties in more detail, we directed our attention towards proteins as potential binding-targets for compounds **1**–**3**.

### 2.5. Binding to Model Proteins

In order to evaluate if the synthetized compounds coordinate to biological macromolecules in particular proteins, and to check if the degradation of compound **3** is the result of these interactions, we have studied the formation of potential adducts that could form upon reaction of compounds **1** or **3** with model proteins hen egg white lysozyme (HEWL) and bovine pancreatic ribonuclease (RNase A), which have been extensively used in the past in metalation studies [51,52,53]. First, the stability of the compounds in the presence of the two proteins was evaluated by registering UV-Vis absorption spectra of the compounds, separately, as function of time (Figures S6–S8). Data indicate that in mixed ethanol/aqueous and DMSO/aqueous solutions the compounds remain stable, with only minor precipitation occurring after 24 h. Then, fluorescence spectra of the two compounds alone and in the presence of HEWL were collected in mixed ethanol/aqueous solutions, upon irradiation at 375 nm, which selectively excites assayed compounds (Figure S9). Interestingly, comparison of the spectra collected upon excitation at 375 nm reveals that ferrocenyl derivative **3** becomes emissive in the presence of HEWL (Figure S9). To verify that this emission is due to the formation of an adduct of **3** with the protein, electrospray ionization mass spectrometry (ESI-MS) spectra of the preformed HEWL-compound **3** adduct were measured and the variations of the HEWL fluorescence in the presence of compounds **1** and **3** were analyzed. To further confirm the binding of compound **3** to the protein, surface plasmon resonance (SPR) measurements were also collected.

**Figure 8:**
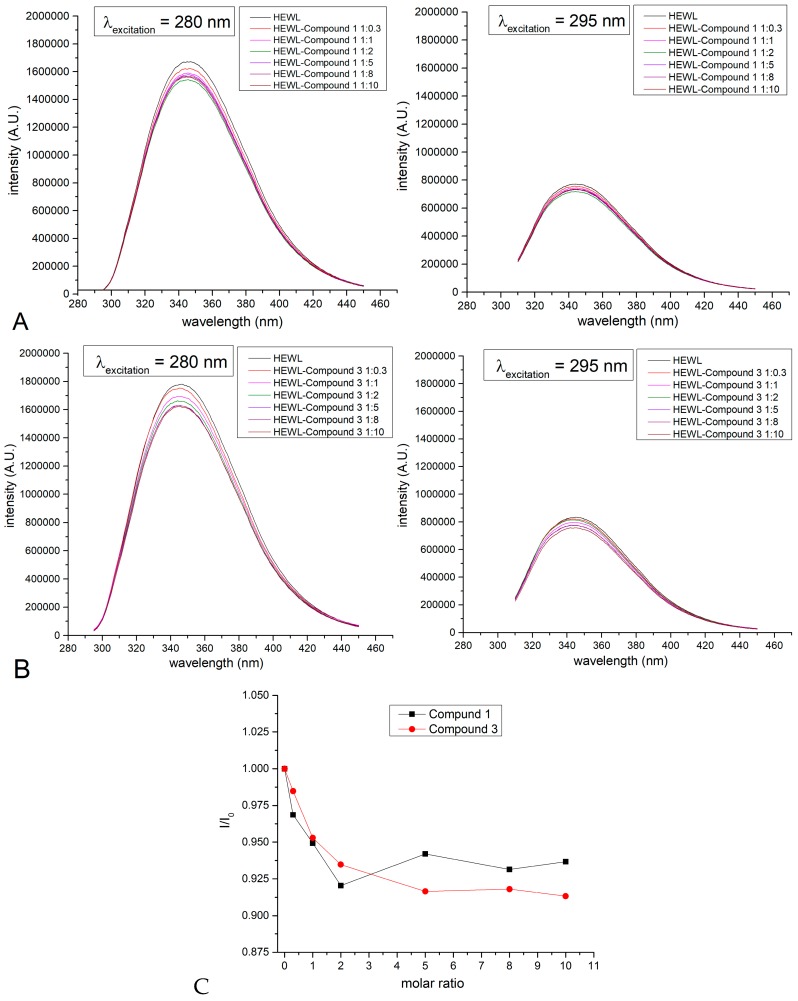
Fluorescence spectra of hen egg white lysozyme (HEWL) adducts with compounds **1** and **3** (0.05 mg/mL in 10% ethanol), prepared by mixing the protein with increasing concentrations of assayed compounds. (**A**) spectra of HEWL and **1**; (**B**) spectra of HEWL and **3**; (**C**) *I*/*I*o as a function of protein to compound ratio. Spectra were collected upon excitation at 280 nm (left panel) and 295 nm (right panel).

In particular, fluorescence spectra of HEWL in the presence of different amounts of the two compounds, separately, upon excitation at 280 and 295 nm, were registered using samples prepared by mixing the protein with the two compounds separately at different protein to compound ratios, and then dialyzing to remove the excess of the assayed compound (Figure 8). Although limited, the observed quenching of fluorescence intensities at increased concentrations of the compounds corroborate the formation of “Re-protein” adducts.

Further indications of the binding of compound **3** to HEWL were obtained by SPR data, reported in Figure S10, and performed on immobilized HEWL protein employing compound **3** as analyzed. Although limited by poor water solubility of the compound that fails to reach saturated binding conditions and to precisely estimate the dissociation constant, these experiments indicate a value of *K*_D_ at least in the high micromolar range.

**Figure 9:**
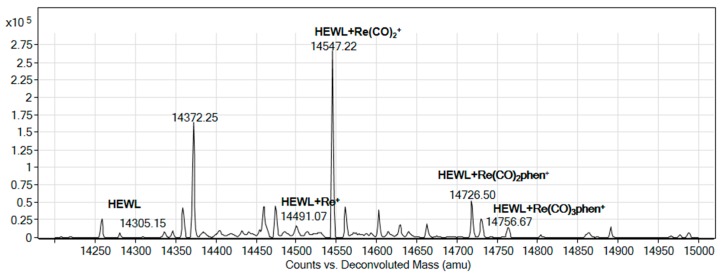
Deconvoluted electrospray ionization mass spectrometry (ESI-MS) spectra of HEWL (1 × 10^−4^ M) incubated for 24 h at room temperature in the presence of compound **3**. Experimental conditions: protein to metal ratio 1:10, 20 mM ammonium acetate buffer, pH 6.8.

The ESI-MS spectrum of the adduct formed in the reaction of HEWL and compound **3** in a 1:10 protein to compound molar ratio is reported in Figure 9. The spectrum features new peaks at a molecular weight higher than that of the protein.

These peaks were assigned to adducts of HEWL with [Re(CO)_3_(phen)]^+^, [Re(CO)_2_(phen)]^+^ and [Re(CO)_2_]^+^ fragments, respectively, and suggest post-dissociative binding of **3** to HEWL molecule.

## 3. Experimental Section

### 3.1. General Information

All preparations were carried out using standard Schlenk techniques. Chromatographic separations were carried out using aluminium oxide (EcoChrom^TM^ MP Biomedicals, Santa Ana, CA, USA). THF was distilled over Na/benzophenone prior to use. Other solvents were of reagent grade and were used without prior purification. The NMR spectra were recorded on a Bruker AV600 Kryo (600 MHz) spectrometer (Bruker, Bremen, Germany). Chemical shifts are reported in δ (ppm) using residual DMSO (^1^H δ 2.50 ppm) as references. Mass spectra were recorded using LSIMS methods on a Finnigan MAT 95 mass spectrometer (Thermo Finnigan MAT GmbH, Bremen, Germany). IR spectra were recorded on a FTIR Nexus Nicolet apparatus (Nicolet, Madison, WI, USA). Microanalyses were determined by the Analytical Services of the Polish Academy of the Sciences (Łódź, Poland).

### 3.2. Chemistry

#### Synthesis of Complexes **1**–**3**

General procedure for the synthesis of fac-[Re(CO)_3_(1,10-phenantroline)L] L = heptanoic acid, hept-6-ynoic acid and 4-(1-ferrocenyl)-butanoic acid **1**, **2**, **3** complexes:

Trimethylamine (46 mg, 0.045 mmol, 63 μL) and silver trifluoromethanesulfonate (116 mg, 0.45 mmol) were added to a stirred mixture of the acid (0.3 mmol) and *fac*-[Re(CO)_3_(1,10-phenantroline)Cl] (220 mg, 0.45 mmol) in THF (60 mL). The reaction mixture was stirred under argon atmosphere at 70 °C for 18 h (for **1** and **3**) or 72 h (for **2**). The solvent was evaporated to dryness to afford a solid which was subjected to column chromatography on deactivated Al_2_O_3_ (Al_2_O_3_/H_2_O 30 g: 1.25 g) with dichloromethane as eluent. Crystallization from dichloromethane/*n*-hexane gave pure products.

**1** yellow solid, 95% (165 mg). ^1^H-NMR (600 MHz, DMSO-*d*_6_): δ = 9.43 (dd, *J*_H,H_ = 5.4 Hz, 1.2 Hz, 2H, phen), 8.96 (dd, *J*_H,H_ = 8.4 Hz, 1.2 Hz, 2H, phen), 8.31 (s, 2H, phen), 8.09 (dd, *J*_H,H_ = 8.4 Hz, 5.4 Hz, 2H, phen), 1.45 (t, *J*_H,H_ = 7.2 Hz, 2H, CH_2_), 0.82 (sext, *J*_H,H_ = 7.2 Hz, 2H, CH_2_), 0.66 (t, *J*_H,H_ = 7.2 Hz, 3H, CH_3_), 0.59–0.50 (m, 4H, CH_2_), 0.31 (quint, *J*_H,H_ = 7.2 Hz, 2H, CH_2_). ^13^C{^1^H} NMR (150 MHz, DMSO-*d*_6_): δ = 198.5, 194.7, 176.3, 154.0, 146.0, 139.4, 130.4, 127.7, 126.3, 35.9, 30.9, 27.8, 25.6, 21.8, 13.9. LSI MS: *m*/*z* = 581 (M + H^+^). FTIR (KBr ν [cm^−1^]): 2955 (C-H), 2930 (C-H), 2854 (C-H), 2027 (Re-C≡O), 1917 (Re-C≡O), 1869 (Re-C≡O), 1622 (C=O) cm^−1^. Anal. Calcd. for C_22_H_21_N_2_O_5_Re: C, 45.59%; H, 3.65%; N, 4.83%. Found: C, 45.74%; H, 3.75%; N, 5.02%.

**2** yellow solid, 65% (112 mg). ^1^H-NMR (600 MHz, DMSO-*d*_6_): δ = 9.43 (dd, *J*_H,H_ = 5.1Hz, 1.2 Hz, 2H, phen), 8.95 (dd, *J*_H,H_ = 7.8 Hz, 1.2 Hz, 2H, phen), 8.03 (s, 2H, phen), 8.09 (dd, *J*_H,H_ = 8.4 Hz, 5.1 Hz, 2H, phen), 2.56 (t, *J*_H,H_ = 2.4 Hz, 1H, C≡CH), 1.49–1.45 (m, 4H, CH_2_), 0.66 (quint, *J*_H,H_ = 7.2 Hz, 2H, CH_2_), 0.50 (quint, *J*_H,H_ = 7.2 Hz, 2H, CH_2_). ^13^C{^1^H} NMR (150 MHz, DMSO-*d*_6_): δ = 198.5, 194.7, 176.1, 154.1, 154.0, 146.0, 139.5, 130.4, 127.8, 126.3, 84.1, 71.0, 35.4, 27.0, 24.8, 17.2. LSI MS: *m*/*z* = 576 (M + H^+^). FTIR (KBr ν [cm^−1^]): 2955 (C-H), 2927 (C-H), 2854 (C-H), 2119 (C≡C), 2024 (Re-C≡O), 1910 (Re-C≡O), 1866 (Re-C≡O), 1606 (C=O) cm^−1^. Anal. Calcd. for C_22_H_17_N_2_O_5_Re: C, 45.91%; H, 2.98%; N, 4.87%. Found: C, 45.93%; H, 3.00%; N, 5.15%.

**3** orange solid, 48% (50 mg). ^1^H-NMR (600 MHz, DMSO-*d*_6_): δ = 9.45 (dd, *J*_H,H_ = 5.1 Hz, 1.2 Hz, 2H, phen), 8.96 (dd, *J*_H,H_ = 8.1 Hz, 1.2 Hz, 2H, phen), 8.30 (s, 2H, phen), 8.10 (dd, *J*_H,H_ = 8.1 Hz, 5.1 Hz, 2H, phen), 3.92 (s, 5H, C_5_H_5_), 3.87 (pt, *J*_H,H_ = 1.8 Hz, 2H, C_5_H_4_), 3.53 (pt, *J*_H,H_ = 1.8 Hz, 2H, C_5_H_4_), 1.49 (t, *J*_H,H_ = 7.2 Hz, 2H, CH_2_), 1.41 (t, *J*_H,H_ = 7.8 Hz, 2H, CH_2_), 0.87 (quint, *J*_H,H_ = 7.2 Hz, 2H, CH_2_). ^13^C{^1^H} NMR (150 MHz, DMSO-*d*_6_): δ = 198.5, 194.6, 176.3, 154.0, 146.0, 139.5, 130.4, 127.8, 126.3, 88.4, 68.1, 67.4, 66.5, 35.6, 27.6, 26.7. LSI MS: *m*/*z* = 722 (M^+^). FTIR (KBr ν [cm^−1^]): 3085(C-H), 2955(C-H), 2933(C-H), 2015(Re-C≡O), 1910(Re-C≡O), 1879(Re-C≡O), 1616(C=O) cm^−1^. Anal. Calcd. for C_29_H_23_N_2_O_5_ReFe: C, 48.27%; H, 3.21%; N, 3.88%. Found: C, 48.27%; H, 3.32%; N, 4.02%.

### 3.3. Quantum Chemical Computations

The molecular geometry of **1** was optimized using the hybrid gradient corrected correlation functional B3LYP [54]. For C, H, N, and O the Ahlrichs split-valence basis set SVP [55] was applied and for valence orbitals of Re the quadruple-dzeta quality atomic basis set QZVP [56] was applied, respectively. The inner-core electrons of Re were substituted with a relativistic effective core potential [57]. Five lowest-energy electronic transitions were calculated for the DFT optimized ground-state geometry using the time-dependent density functional theory (TD-DFT) and applying the same basis sets as for the geometry optimization. All computations were carried out using the Gaussian 09 computer program package [58].

### 3.4. X-ray Crystallography

Good quality single-crystals of **1**–**3** were selected for the X-ray diffraction experiments at *T* = 100(2) K. They were mounted with paratone-N oil at the MiTeGen micro-mounts. Diffraction data were collected on the Agilent Technologies SuperNova Dual Source diffractometer with Mo*K*α radiation (*λ* = 0.71073 Å) using CrysAlis RED software (CrysAlis CCD and CrysAlis RED, Oxford Diffraction, Oxford Diffraction Ltd.: Yarnton, UK, 2008). The analytical numerical absorption correction using a multifaceted crystal model based on expressions derived by R.C. Clark & J.S. Reid [59] (**1** and **2**) and numerical absorption correction based on gaussian integration over a multifaceted crystal model (**3**) implemented in SCALE3 ABSPACK scaling algorithm, were applied [60]. The structural determination procedure was carried out using the SHELX package [61]. The structures were solved with direct methods and then successive least-square refinement was carried out based on the full-matrix least-squares method on *F*^2^ using the XLMP program [61]. All H-atoms were positioned geometrically, with C–H equal to 0.93, 0.96 and 0.97 Å for the aromatic/methylidyne, methyl and methylene H-atoms, respectively, and constrained to ride on their parent atoms with U_iso_(H) = x*U*_eq_(C), where x = 1.2 for the aromatic, methylidyne and methylene H-atoms, and x = 1.5 for the methyl H-atoms. The figures for this publication were prepared using *ORTEP-3* program [62].

### 3.5. Luminescence Measurements

UV-Vis absorption spectra were recorded with a Varian Cary 300 double beam spectrometer. Luminescence spectra were measured for air-saturated and degassed diluted (*c* ≈ 5 × 10^−5^ M) solutions in ethanol with a Horiba Jobin Yvon Fluorolog 3 steady-state fluorescence spectrometer. For decay time measurements, a PicoQuant LDH-P-C-375 pulsed diode laser (λ_exc_ = 372 nm, pulse width 100 ps) was applied as the excitation source. The emission signal was detected with a cooled photomultiplier attached to a FAST ComTec, München, Germany, multichannel scalar card with a time resolution of 250 ps. Photoluminescence quantum yields φ_PL_ were determined with a Hamamatsu C9920-02 system equipped with a Spectralon^®^ integrating sphere.

### 3.6. Biology

#### 3.6.1. Biological Imaging

Human HeLa 21.4 cells were cultured for 48 h after seeding in Petri dishes with a glass bottom (MaTek), reaching approximately 50% confluency. The cells were grown in Dulbeco’s minimal essential medium (DMEM) with 5% FCS, at 5% CO_2_ and 37 °C. The same medium was used to perform all microscopy experiments. Directly before imaging, the cells were incubated with 200 nM of **1**, **2** or **3** added to the imaging medium. Stock solutions of these compounds were freshly prepared in DMSO (200 µM). Additionally, mitochondria of live cells were labelled (where indicated) with MitoTracker^®^ Green (Thermofisher, Poland) by incubation with 100 nM of the dye for 20 min. Alternatively, cells were incubated with 20 µM of dihydroethidium (DHE) for 15 min, to detect production of reactive oxygen species (ROS). Where indicated, mitochondrial electric potential was dispersed [63] by incubation with 5 µM carbonyl cyanide-*p*-trifluoromethoxyphenylhydrazone (FCCP) (30 min) prior to loading with **2**. Loss of plasma membrane integrity (indicator of cell viability) was monitored using staining of cell nuclei with propidium iodide (PI), as described before [50]. The imaging was performed by using LSM 780 confocal system (Zeiss), equipped AxioObserver Z1 inverted microscope, 63× oil immersion objective (NA 1.4), 405 diode laser (30 mW), 488 nm Ar laser (25 mW) a multi-anode PMT (32 elements). Luminescence spectrum was registered from single confocal sections (pinhole set to 1 Airy unit), in 410 nm–685 nm range, with 8.5 nm spectral precision, using 405 nm (1.8%) excitation. Where indicated, detector elements were combined into detection bands corresponding to 600–700 nm (**1**, **2**, **3**, excitation 405 nm) and 500–550 nm (MitoTracker^®^ Green, excitation 488 nm). The luminescence and transmitted light images were collected with 8.4 µs pixel dwell time and pixel size of 0.176 µm. Alternatively, the imaging was performed using Leica SP8 confocal system, equipped with a DMI6000 inverted microscope, 63× oil immersion objective (NA 1.4), 405 nm pulsed (40 MHz) diode laser (5 mW nominal power), 488 nm Ar laser (30 mW), hybrid (HyD) and conventional (PMT) detectors. Luminescence (**1**, **2**, **3**) was excited using the 405 laser (5%) and registered (PMT) from single confocal sections (pinhole set to 1 Airy unit) in 600–700 nm range. Fluorescence of ethidium (product of DHE oxidation by ROS) was excited with 488 nm light and detected in 550–585 nm range with HyD (integration mode). The luminescence and transmitted light images were collected with the speed of 100 lines/s and pixel size of 0.18 µm. Loss of cell viability in the presence of **2** (200 nM) was monitored in time with image cytometry. Image registration was performed with Leica DMI6000 inverted wide-field microscope, equipped 40× dry objective (NA 0.75), 100 W metal halide arc lamp and a 1.4 Mp CCD camera (Sony ICX 285 chip, Leica, Wetzlar, Germany). The HeLa cells were cultured (~50% confluency) and imaged in standard 24-well plates. Transmitted light and fluorescence (580–630 nm range, PI) images were registered for 18 h, with 1 h frame rate. Each well corresponded to 9 (3 × 3 tile) fields of view probed, which translated to at least 220 monitored cells. To quantify phototoxic effects of **2**, the HeLa cells were irradiated with 35 µW of UV light (340–380 nm) for 360 s before onset of the imaging. The time (LT50) corresponding to 50% of imaged cells losing their plasma membrane integrity (manifested by PI staining of nuclei) was quantified for the populations of control (1720) and UV-irradiated (1930) cells, stained with **2**.

#### 3.6.2. Reaction with Model Proteins

Interactions between compounds **1** and **3** and model proteins hen egg white lysozyme (HEWL) and bovine pancreatic ribonuclease (RNase A) were studied by collecting (1) UV-Vis absorption spectra of the two compounds in mixed DMSO/aqueous and ethanol/aqueous solutions in the presence of the two proteins, separately, for 24 h; (2) Intrinsic fluorescence spectra of HEWL samples prepared by incubating the protein in the presence of the compounds at different concentrations for 24 h at room temperature and then dialyzing to remove the unbound compound. Spectra were registered upon excitation at 280, 295 and 375 nm; (3) electrospray ionization mass spectra (ESI MS) of HEWL in the absence and in the presence of the two compounds, separately, upon 24 h of incubation at room temperature; (4) surface plasmon resonance measurements of the formation of HEWL-compound **3** system. UV-Vis absorption spectra were recorded with a Varian Cary 5000 Uv-vis-NIR spectrophotometer under the following experimental conditions: 100% ethanol, 50% ethanol and 50% PBS pH 7.4, 10% ethanol and 90% PBS pH 7.4 in the presence and in the absence of the two proteins. Other spectra were recorded using DMSO instead of ethanol. A Horiba Scientific Fluoromax-4 spectrofluorometer was used to measure fluorescence spectra. Experimental details: excitation wavelengths = 280, 295 and 375 nm; protein concentration = 0.05 mg/mL (0.0035 mM). Spectra were collected in 10% ethanol at 20 °C by comparing the protein emission spectrum with that of the metal complex under the same experimental conditions. Different protein to metal ratio were analyzed (HEWL:complex 1:0.5, 1:1, 1:2, 1:3, 1:5, 1:8, 1:10).

ESI MS measurements were carried out using an LCQ DECA XP Ion Trap mass spectrometer (ThermoElectron, Milan, Italy) equipped with an OPTON ESI source (operating at 4.2-kV needle voltage and 320 °C) in positive mode. Multicharge spectra were deconvoluted using the BioMass program implemented in the Bioworks 3.1 package provided by the manufacturer. In these experiments, proteins were incubated in the presence of the two compounds for 24 h at room temperature in 1:10 protein to metal ratio, 20 mM ammonium acetate pH 6.8.

Surface Plasmon Resonance (SPR) experiments were performed at 25 °C on a Biacore 3000 instrument (GE Healthcare, Buckinghamshire, UK). HEWL was immobilized at 1095 RU, on a CM5 Biacore sensor chip in 10 mM sodium acetate pH 5.5, by using the EDC/NHS chemistry, with a flow rate of 5 μL × min^−1^ and an injection time of 7 min. Binding assays were carried out by injecting 90 µL of analyte, at 20 µL × min^−1^. Experiments were carried out at Ph = 7.4 using HBS and 8% EtOH (*v*/*v*). Solvent correction was performed. Stock solutions of compound **3** at 3.0 mM in EtOH were freshly prepared.

#### 3.6.3. Deposited Data

Cif files of the structure determinations were deposited at the Cambridge Crystallographic Data Centre (CCDC numbers 1524287-1524289). These data can be obtained free of charge via www.ccdc.cam.ac.uk/structures.

## 4. Conclusions

We have synthetized and fully characterized three new neutral *fac*-[Re(CO)_3_(phen)L] compounds (**1**–**3**) with phen = 1,10-phenanthroline and L = O_2_C(CH_2_)_5_CH_3_, O_2_C(CH_2_)_4_C≡CH, and O_2_C(CH_2_)_3_((C_5_H_5_)Fe(C_5_H_4_)). Compounds **1** and **2** display orange luminescence characteristics for the *fac*­[Re(CO)_3_(phen)]^+^ core, whereas **3** is not emissive due to the presence of the ferrocenyl moiety that quenches the emission. Confocal microscopy study reveals selective accumulation of the three compounds in mitochondria. Emission of **3** can only be possible when ferrocene-containing ligand dissociates from the metal to produce another species containing the luminescent *fac*­[Re(CO)_3_(phen)]^+^ core. Thus, dissociation of the ligands from the metal is the basis of the mitochondrial accumulation by these compounds. In particular, the following mechanism is proposed: (i) ancillary carboxylate ligands in **1**–**3** dissociate inside the cell, producing luminescent *fac*­[Re(CO)_3_(phen)]^+^ products; (ii) these cationic species rapidly react with various cellular matrix molecules giving secondary products, which are uptaken into the negatively charged mitochondrial membranes; (iii) follow up reactions lead to covalent (or charge independent) mitochondria binding as confirmed by FCCP experiments. Corroboration of this mechanism comes from in vitro studies of reactions of the compounds **1**–**3** with proteins, which indicate that the *fac*­[Re(CO)_3_(phen)]^+^ entity can bind to model proteins HEWL and RNase A. When accumulated in mitochondria, *fac*­[Re(CO)_3_(phen)]^+^ core triggers elevated ROS production and induces a photocytotoxic effect. 

In summary, our results reveal an interesting dissociation-driven mechanism of biological activity for neutral *fac*-[Re(CO)_3_(phen)L] compounds. We are currently looking for biologically more relevant carboxylate ligands (L = drugs, peptides, natural products) in order to control the biological action profile of *fac*-[Re(CO)_3_(phen)L] complexes. 

## Figures and Tables

**Table 1:** Selected bond distances (Å) and angles (°) of **1**–**3** (Cp_sub_ = substituted Cp ring).

	1	2	3A	3B
Bond distances				
C1-O1	1.154(2)	1.151(5)	1.164(4)	1.174(5)
C2-O2	1.155(2)	1.152(4)	1.160(4)	1.167(5)
C3-O3	1.164(2)	1.165(4)	1.157(4)	1.152(4)
Re1-C1	1.922(2)	1.924(4)	1.903(4)	1.901(4)
Re1-C2	1.923(2)	1.926(3)	1.917(3)	1.911(4)
Re1-C3	1.900(2)	1.903(3)	1.910(3)	1.919(4)
Re1-N1	2.176(2)	2.173(3)	2.177(3)	2.181(3)
Re1-N2	2.184(2)	2.185(3)	2.175(3)	2.180(3)
Re1-O4	2.142(2)	2.146(2)	2.125(2)	2.129(2)
Fe-mid point (Cp_sub_)	-	-	1.654(2)	1.649(2)
Fe-mid point (Cp)	-	-	1.651(2)	1.651(2)
bond angles				
O4-C16-O5	125.33(16)	125.0(3)	125.7(3)	125.5(3)
O4-Re1-N1	81.42(5)	80.83(9)	82.33(9)	79.74(10)
O4-Re1-C1	95.58(7)	94.89(13)	96.39(12)	98.50(13)
O4-Re1-C3	174.96(6)	174.70(12)	174.35(12)	173.61(12)

**Table 2:** Photophysical data for complexes **1**–**3** at ambient temperature in ethanol.

Complex	Absorption Maximum λ_abs_ (nm) (Molar Absorptivity ε (M^−1^ cm^−1^))	Emission Maximum λ_em_ (nm)	Emission Decay Time Τ (ns) ^a^
**1**	375 (4600), 262 (27,600)	630	60
**2**	375 (3600), 262 (24,100)	630	60
**3**	375 (4400), 262 (29,000)	-	-

^a^ measured for degassed solutions.

## Supplementary Materials

Supplementary materials are available online.

## References

[1] Yersin H., Rausch A.F., Czerwieniec R., Hofbeck T., Fischer T. (2011). The triplet state of organo-transition metal compounds. Triplet harvesting and singlet harvesting for efficient OLEDs. Coord. Chem. Rev..

[2] Zhao Q., Li F., Huang C. (2010). Phosphorescent chemosensors based on heavy-metal complexes. Chem. Soc. Rev..

[3] Ashford D.L., Gish M.K., Vannucci A.K., Brennaman M.K., Templeton J.L., Papanikolas J.M., Meyer T.J. (2015). Molecular Chromophore–Catalyst Assemblies for Solar Fuel Applications. Chem. Rev..

[4] Fernández-Moreira V., Thorp-Greenwood F.L., Coogan M.P. (2010). Application of d6 transition metal complexes in fluorescence cell imaging. Chem. Commun..

[5] Baggaley E., Weinstein J.A., Williams J.A.G. (2012). Lighting the way to see inside the live cell with luminescent transition metal complexes. Coord. Chem. Rev..

[6] Lo K.K.-W. (2015). Luminescent Rhenium(I) and Iridium(III) Polypyridine Complexes as Biological Probes, Imaging Reagents, and Photocytotoxic Agents. Acc. Chem. Res..

[7] Thorp-Greenwood F.L. (2012). An Introduction to Organometallic Complexes in Fluorescence Cell Imaging: Current Applications and Future Prospects. Organometallics.

[8] Coogan M.P., Fernández-Moreira V. (2014). Progress with, and prospects for, metal complexes in cell imaging. Chem. Commun..

[9] Lo K.K.-W., Choi A.W.-T., Law W.H.-T. (2012). Applications of luminescent inorganic and organometallic transition metal complexes as biomolecular and cellular probes. Dalton Trans..

[10] Ma D.-L., He H.-Z., Leung K.-H., Chan D.S.-H., Leung C.-H. (2013). Bioactive Luminescent Transition-Metal Complexes for Biomedical Applications. Angew. Chem. Int. Ed..

[11] Gasser G., Ott I., Metzler-Nolte N. (2011). Organometallic anticancer compounds. J. Med. Chem..

[12] Patra M., Gasser G. (2012). Organometallic Compounds: An Opportunity for Chemical Biology?. ChemBioChem.

[13] Bartholomä M., Valliant J., Maresca K.P., Babich J., Zubieta J. (2009). Single amino acid chelates (SAAC): A strategy for the design of technetium and rhenium radiopharmaceuticals. Chem. Commun..

[14] Clède S., Policar C. (2015). Metal–Carbonyl Units for Vibrational and Luminescence Imaging: Towards Multimodality. Chem. Eur. J..

[15] Giordano P.J., Wrighton M.S. (1979). The nature of the lowest excited state in *fac*-tricarbonylhalobis(4-phenylpyridine)rhenium(I) and *fac*-tricarbonylhalobis(4,4′-bipyridine)rhenium(I): Emissive organometallic complexes in fluid solution. J. Am. Chem. Soc..

[16] Záliš S., Milne C.J., El Nahhas A., Blanco-Rodríguez A.M., van der Veen R.M., Vlček A. (2013). Re and Br X-ray Absorption Near-Edge Structure Study of the Ground and Excited States of [ReBr(CO)_3_(bpy)] Interpreted by DFT and TD-DFT Calculations. Inorg. Chem..

[17] Czerwieniec R., Kapturkiewicz A., Lipkowski J., Nowacki J. (2005). Re(I)(tricarbonyl)^+^ complexes with the 2-(2-pyridyl)-*N*-methyl-benzimidazole, 2-(2-pyridyl)benzoxazole and 2-(2-pyridyl)benzothiazole ligands—Syntheses, structures, electrochemical and spectroscopic studies. Inorg. Chim. Acta.

[18] Kirgan R.A., Sullivan B.P., Rillema D.P. (2007). Photochemistry and photophysics of coordination compounds: Rhenium. Top. Curr. Chem..

[19] Stephenson K.A., Banerjee S.R., Besanger T., Sogbein O.O., Levadala M.K., McFarlane N., Lemon J.A., Boreham D.R., Maresca K.P., Brennan J.D. (2004). Bridging the Gap between in Vitro and in Vivo Imaging:  Isostructural Re and 99mTc Complexes for Correlating Fluorescence and Radioimaging Studies. J. Am. Chem. Soc..

[20] Amoroso A.J., Arthur R.J., Coogan M.P., Court J.B., Fernández-Moreira V., Hayes A.J., Lioyd D., Millet C., Pope S.J.A. (2008). 3-Chloromethylpyridyl bipyridine fac-tricarbonyl rhenium: A thiol-reactive luminophore for fluorescence microscopy accumulates in mitochondria. New. J. Chem..

[21] Fernández-Moreira V., Thorp-Greenwood F.L., Amoroso A.J., Cable J., Court J.B., Gray V., Hayes A.J., Jenkins R.J., Kariuki B.M., Lloyd D. (2010). Uptake and localisation of rhenium *fac*-tricarbonyl polypyridyls in fluorescent cell imaging experiments. Org. Biomol. Chem..

[22] Balasingham R.G., Thorp-Greenwood F.L., Williams C.F., Coogan M.P., Pope S.J.A. (2012). Biologically Compatible, Phosphorescent Dimetallic Rhenium Complexes Linked through Functionalized Alkyl Chains: Syntheses, Spectroscopic Properties, and Applications in Imaging Microscopy. Inorg. Chem..

[23] Langdon-Jones E.E., Symonds N.O., Yates S.E., Hayes A.J., Lloyd D., Williams R., Coles S.J., Horton P.N., Pope S.J.A. (2014). Fluorescent Rhenium-Naphthalimide Conjugates as Cellular Imaging Agents. Inorg. Chem..

[24] Clède S., Lambert F., Saint-Fort R., Plamont M.-P., Bertrand H., Vessières A., Policar C. (2014). Influence of the Side-Chain Length on the Cellular Uptake and the Cytotoxicity of Rhenium Triscarbonyl Derivatives: A Bimodal Infrared and Luminescence Quantitative Study. Chem. Eur. J..

[25] Ferri E., Donghi D., Panigati M., Prencipe G., D’Alfonso L., Zanoni I., Baldoli C., Maiorana S., D’Alfonso G., Licandro E. (2010). Luminescent conjugates between dinuclear rhenium(I) complexes and peptide nucleic acids (PNA) for cell imaging and DNA targeting. Chem. Commun..

[26] Funkhouser J. (2002). Reintroducing pharma: Theranostic revolution. Curr. Drug Discov..

[27] Celli J.P., Spring B.Q., Rizvi I., Evans C.I., Samkoe K.S., Verma S., Pogue B.W., Hasan T. (2010). Imaging and Photodynamic Therapy: Mechanisms, Monitoring, and Optimization. Chem. Rev..

[28] Juarranz A., Jaèn P., Sanz-Rodriguez F., Cuevas J., González S. (2008). Photodynamic therapy of cancer. Basic principles and applications. Clin. Transl. Oncol..

[29] Taub A.F. (2007). Photodynamic Therapy: Other Uses. Dermatol. Clin..

[30] Lo K.K.-W., Louie M.-W., Sze K.-S., Lau J.S.-Y. (2008). Rhenium(I) Polypyridine Biotin Isothiocyanate Complexes as the First Luminescent Biotinylation Reagents:  Synthesis, Photophysical Properties, Biological Labeling, Cytotoxicity, and Imaging Studies. Inorg. Chem..

[31] Louie M.-W., Liu H.-W., Lam M.H.-C., Lam Y.-W., Lo K.K.-W. (2011). Luminescent Rhenium(I) Polypyridine Complexes Appended with an α-d-Glucose Moiety as Novel Biomolecular and Cellular Probes. Chem. Eur. J..

[32] Zhang K.Y., Tso K.K.-S., Louie M.-W., Liu H.-W., Lo K.K.-W. (2013). A Phosphorescent Rhenium(I) Tricarbonyl Polypyridine Complex Appended with a Fructose Pendant That Exhibits Photocytotoxicity and Enhanced Uptake by Breast Cancer Cells. Organometallics.

[33] Viola-Villegas N., Rabideau A.E., Cesnavicious J., Zubieta J., Doyle R.P. (2008). Targeting the Folate Receptor (FR): Imaging and Cytotoxicity of ReI Conjugates in FR-Overexpressing Cancer Cells. ChemMedChem.

[34] Viola-Villegas N., Rabideau A.E., Bartholom M., Zubieta J., Doyle R.P. (2009). Targeting the Cubilin Receptor through the Vitamin B12 Uptake Pathway: Cytotoxicity and Mechanistic Insight through Fluorescent Re(I) Delivery. J. Med. Chem..

[35] Kitanovic I., Can S., Alborzinia H., Kitanovic A., Pierroz V., Leonidova A., Pinto A., Spingler B., Ferrari S., Molteni R. (2014). A Deadly Organometallic Luminescent Probe: Anticancer Activity of a ReI Bisquinoline Complex. Chem. Eur. J..

[36] Leonidova A., Pierroz V., Rubbiani R., Heier J., Ferrari S., Gasser G. (2014). Towards cancer cell-specific phototoxic organometallic rhenium(I) complexes. Dalton. Trans..

[37] Leonidova A., Pierroz V., Rubbiani R., Lan Y., Schmitz A.G., Kaech A., Sigel R.K.O., Ferrari S., Gasser G. (2014). Photo-induced uncaging of a specific Re(I) organometallic complex in living cells. Chem. Sci..

[38] Frey-Forgues S., Delavaux-Nicot B. (2000). Ferrocene and ferrocenyl derivatives in luminescent systems. J. Photochem. Photobiol. A Chem..

[39] Feliz M., Ferraudi G. (1998). Charge-Transfer Processes in (4-Nitrobenzoate)Re(CO)_3_(azine)_2_ Complexes. Competitive Reductions of 4-Nitrobenzoate and Azine in Thermally and Photochemically Induced Redox Processes. Inorg. Chem..

[40] Juliarena M.P., Ruiz G.T., Wolcan E., Lezna R.O., Feliz M.R., Ferraudi G., Guerrero J. (2007). Inhibition of the *fac*-(RCO_2_)ReI(CO_3_)(bis-azine) Photodecarboxylation of the Carboxylate Ligand, RCO_2_-, When R Is a Strongly Electron Donating Group:  Thermal and Photochemical Properties of Complexes Where R = Ferrocene, 4-(Dimethylamino)benzyl. Organometallics.

[41] Juliarena M.P., Lezna R.O., Ruiz G.T., Féliz M.R., Ferraudi G., Wolcan E. (2008). On the nature of the redox products of 4-((CH_3_)_2_N)C_6_H_4_–CO_2_−and 4-((CH_3_)_2_N)C_6_H_4_–CO_2_–Re(CO)_3_(1,10-phenanthroline): A spectroelectrochemical, pulse radiolysis and flash photochemical study. Polyhedron.

[42] Cuesta L., Hevia E., Morales D., Pérez J., Riera L., Miguel D. (2006). Reactivity of Molybdenum and Rhenium Hydroxo Complexes toward Organic Electrophiles:  Reactions that Afford Carboxylato Products. Organometallics.

[43] Maggioni D., Fenili F., D’Alfonso L., Donghi D., Panigati M., Zanoni I., Marzi R., Manfredi A., Ferruti P., D’Alfonso G. (2012). Luminescent Rhenium and Ruthenium Complexes of an Amphoteric Poly(amidoamine) Functionalized with 1,10-Phenanthroline. Inorg. Chem..

[44] Chu W.-K., Ko C.-C., Chan K.-C., Yiu S.-M., Wong F.-L., Lee C.-S., Roy V.A.L. (2014). A Simple Design for Strongly Emissive Sky-Blue Phosphorescent Neutral Rhenium Complexes: Synthesis, Photophysics, and Electroluminescent Devices. Chem. Mat..

[45] Cuesta L., Huertos M.A., Morales D., Pérez J., Riera L., Riera V., Miguel D., Menéndez-Velázquez A., Garcia-Granda S. (2007). Synthesis, Structure, and Reactivity of Mononuclear Re(I) Oximato Complexes. Inorg. Chem..

[46] Kowalski K., Szczupak Ł., Bernaś T., Czerwieniec R. (2015). Luminescent rhenium(I)–chromone bioconjugate: Synthesis, photophysical properties, and confocal luminescence microscopy investigation. J. Organomet. Chem..

[47] Yamaguchi Y., Ding W., Sanderson C.T., Borden M.L., Morgan M.J., Kutal C. (2007). Electronic structure, spectroscopy, and photochemistry of group 8 metallocenes. Coord. Chem. Rev..

[48] Dunn W.K., Kamocka M.M., McDonald H.J. (2011). A practical guide to evaluating colocalization in biological microscopy. Am. J. Physiol. Cell Physiol..

[49] Benz R., McLaughlin S. (1983). The molecular mechanism of action of the proton ionophore FCCP (carbonylcyanide *p*-trifluoromethoxyphenylhydrazone). Biophys. J..

[50] Bernaś T., Dobrucki J. (2004). Backscattered light confocal imaging of intracellular MTT-formazan crystals. Micro Res. Technol..

[51] Messori L., Marzo T., Gabbiani C., Valdes A.A., Quiroga A.G., Merlino A. (2013). Peculiar Features in the Crystal Structure of the Adduct Formed between *cis*-PtI_2_(NH_3_)_2_ and Hen Egg White Lysozyme. Inorg. Chem..

[52] Messori L., Merlino A. (2014). Cisplatin Binding to Proteins: Molecular Structure of the Ribonuclease A Adduct. Inorg. Chem..

[53] Messori L., Merlino A. (2014). Ruthenium metalation of proteins: The X-ray structure of the complex formed between NAMI-A and hen egg white lysozyme. Dalton Trans..

[54] Becke A.D. (1993). Density-functional thermochemistry. III. The role of exact exchange. J. Chem. Phys..

[55] Schaefer A., Horn H., Ahlrichs R. (1992). Fully optimized contracted Gaussian basis sets for atoms Li to Kr. J. Chem. Phys..

[56] Weigend F., Ahlrichs R. (2005). Balanced basis sets of split valence, triple zeta valence and quadruple zeta valence quality for H to Rn: Design and assessment of accuracy. Phys. Chem. Chem. Phys..

[57] Hay P.J., Wadt W.R. (1985). *Ab initio* effective core potentials for molecular calculations. Potentials for the transition metal atoms Sc to Hg. J. Chem. Phys..

[58] Frisch M.J., Trucks G.W., Schlegel H.B., Scuseria G.E., Robb M.A., Cheeseman J.R., Scalmani G., Barone V., Mennucci B., Petersson G.A. (2009). Gaussian09W, V. 8.0.

[59] Clark R.C., Reid J.S. (1995). The analytical calculation of absorption in multifaceted crystals. Acta Cryst. Sect. A.

[60] (2008). CrysAlis CCD.

[61] Sheldrick G.M. (2008). A short history of SHELX. Acta Cryst. Sect. A.

[62] Farrugia L.J. (1997). ORTEP-3 for Windows—A version of ORTEP-III with a Graphical User Interface (GUI). J. Appl. Cryst..

[63] To M.-S., Aromataris E.C., Castro J., Roberts M.L., Barritt G.J., Rychkov G.Y. (2010). Mitochondrial uncoupler FCCP activates proton conductance but does not block store-operated Ca^2+^ current in liver cells. Arch. Biochem. Biophys..

