# Supplementary material for: Mitochondria Targeting with Luminescent Rhenium(I) Complexes
Source: Molecules. 2017 May 15;22(5):809. doi: 10.3390/molecules22050809 (PMC6154647; doi:10.3390/molecules22050809)
Supplement: Supplementary file 1 [file molecules-22-00809-s001.pdf]

Mitochondria targeting with luminescent rhenium(I) complexes.

Joanna Skiba [1], Tytus Bernaś [2], Damian Trzybiński [3], Krzysztof Woźniak [3], Giarita Ferraro [4], Daniela Marasco [5,6,7], Antonello Merlino [4,7], Marsel Z. Shafikov [8,10], Rafał Czerwieniec [9,10,*] and Konrad Kowalski [1,*]

[1] Faculty of Chemistry, Department of Organic Chemistry, University of Łódź, Tamka 12, 91-403 Łódź, Poland; asiaskiba@02.pl

[2] Nencki Institute of Experimental Biology, Polish Academy of Sciences, ul. Pasteura 3, 02-093 Warszawa, Poland; t.bernas@nencki.gov.pl

[3] Faculty of Chemistry, Biological and Chemical Research Centre, University of Warsaw, Żwirki i Wigury 101, 02-089 Warszawa, Poland; trzybinski@chem.uw.edu.pl (D.T.); kwozniak@chem.uw.edu.pl (K.W.)

[4] Department of Chemical Sciences, University of Naples Federico II, Complesso Univ. di Monte Sant' Angelo, Via Cintia, I-80126, Napoli, Italy, giarita.ferraro@unina.it (G.F.); antonello.merlino@unina.it (A.M.)

[5] Department of Pharmacy, University of Naples Federico II, Via Mezzocannone 16, 80134, Napoli, Italy; daniela.marasco@unina.it (D. M.)

[6] CIRPEB: Centro Interuniversitario di Ricerca sui Peptidi Bioattivi, Via Mezzocannone 16, I-80134, Napoli, Italy; daniela.marasco@unina.it (D. M.)

[7] CNR Institute of Biostructures and Bioimages, Via Mezzocannone 16, I-80134, Napoli, Italy; antonello.merlino@unina.it (A.M.)

[8] Department of Technology of Organic Synthesis, Institute of Chemical Engineering,
Ural Federal University, 19 Mira str., Ekaterinburg, Russia, 620002. shafikoff@gmail.com (M.S.)

[9] Lehrstuhl für Anorganische Chemie I, University of Bayreuth, D-95440 Bayreuth, Germany.

[10] Institut für Physikalische und Theoretische Chemie, Universität Regensburg, Universitätsstraße 31, D-93040 Regensburg, Germany;

* Correspondence: Rafal.Czerwieniec@uni-bayreuth.de (R.C.); kondor15@wp.pl (K.K.);

Supplementary Information

Contents

Figure S1 ^{1}H-NMR spectrum of **1** **S3**

Figure S2 ^{1}H-NMR spectrum of **2** **S4**

Figure S3 ^{1}H-NMR spectrum of **3** **S5**

Figure S4 Confocal image of living HeLa cells stained with **1** and **3**. **S6**

Figure S5 Confocal image of living HeLa cells stained with **2** and incubated with FCCP before staining with **2**. **S7**

Figure S6. UV-Vis spectra of 0.2 mM compound **1** or **3** in 100% DMSO (panels A-C) and 100% ethanol. **S8**

Figure S7 UV-Vis spectra of 0.2 mM compound **1** or **3** in 50% ethanol and 50% PBS pH 7.4 in the absence and in the presence of HEWL and of RNase A. **S10**

Figure S8 UV-Vis spectra of 0.2 mM compound **1** or **3** in 50% DMSO and 50% PBS pH 7.4 in the absence and in the presence of HEWL and of RNase A in a 1 : 3 protein to metal molar ratio. **S11**

Figure S9 Fluorescence spectra of HEWL in the presence of increasing concentration of complex **1** and complex **3** upon excitation at 375nm. **S12**

Figure S10 Sensorgrams for the interaction between compound **3** and HEWL at 50, 100 and 200 μM. **S13**

Table S1 Crystallographic data and structural refinement details of **1–3**. **S14**

Table S2 Bond lengths for **1**. **S15**

Table S3 Valence angles for **1**. **S16**

Table S4 Torsion angles for **1**. **S17**

Table S5 Bond lengths for **2**. **S19**

Table S6 Valence angles for **2**. **S20**

Table S7 Torsion angles for **2**. **S21**

Table S8 Bond lengths for **3**. **S22**

Table S9 Valence angles for **3**. **S24**

Table S10 Torsion angles for **3**. **S27**

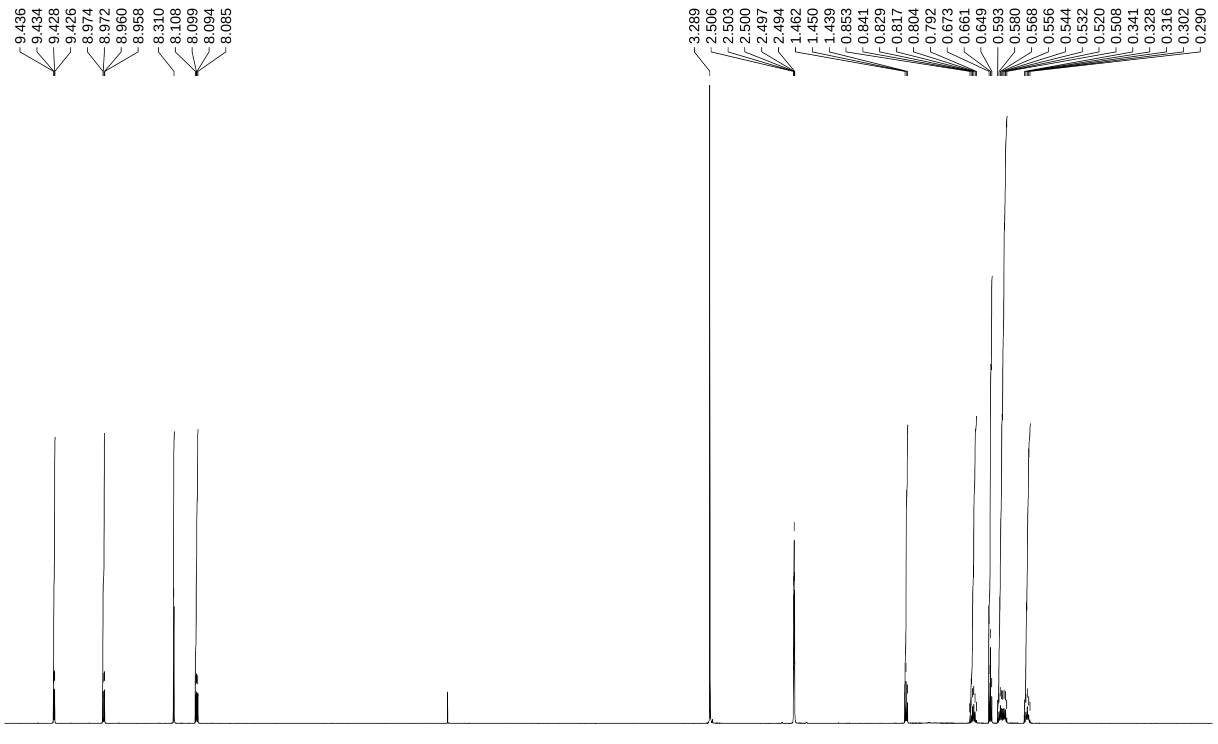

Figure S1 ^{1}H NMR spectrum of **1**

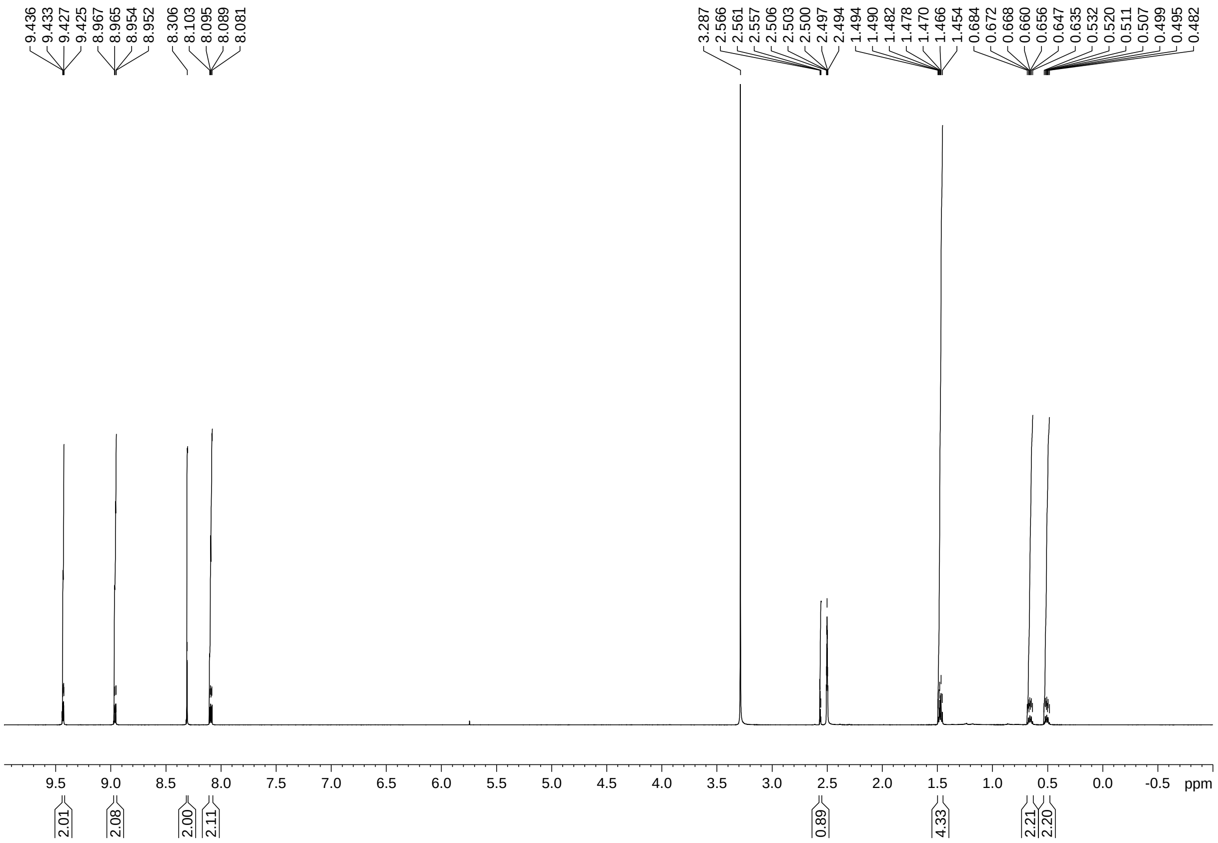

Figure S2 ^{1}H NMR spectrum of **2**

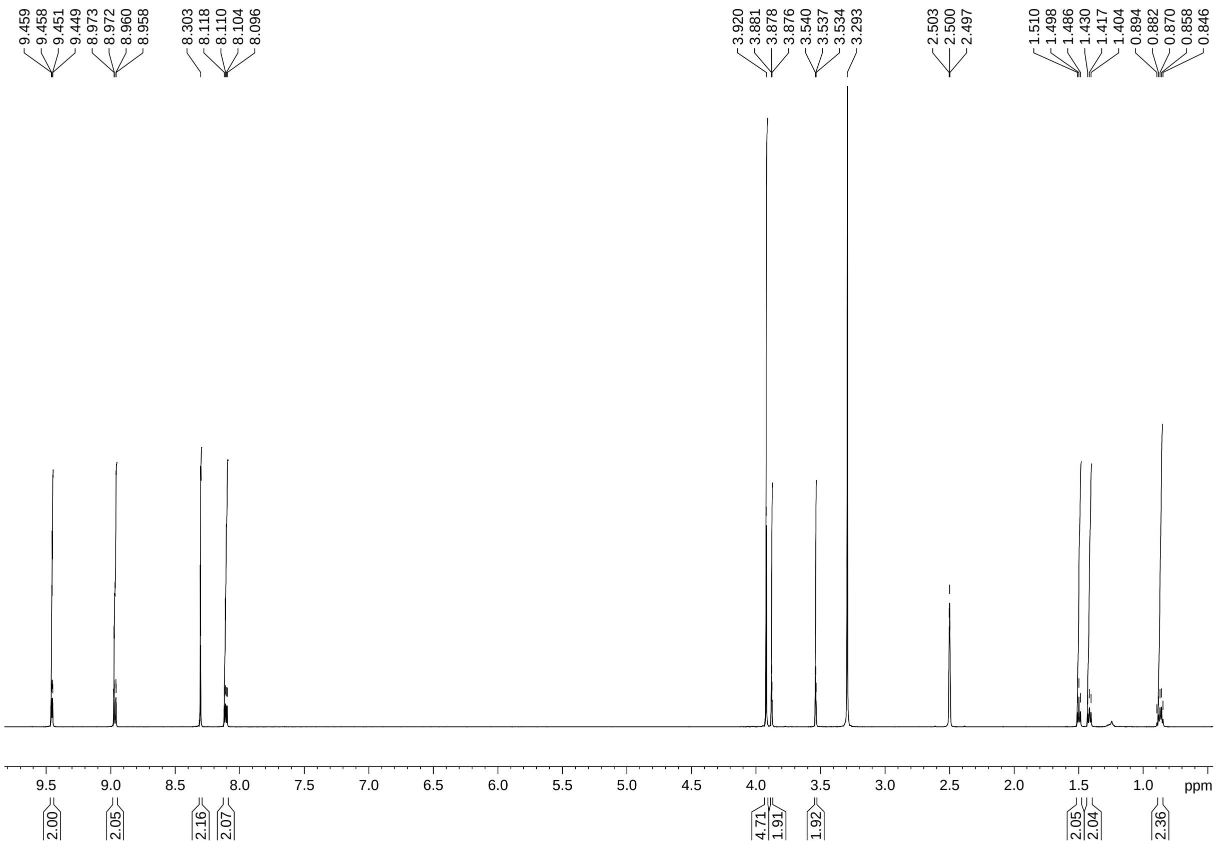

Figure S3 ^{1}H NMR spectrum of **3**

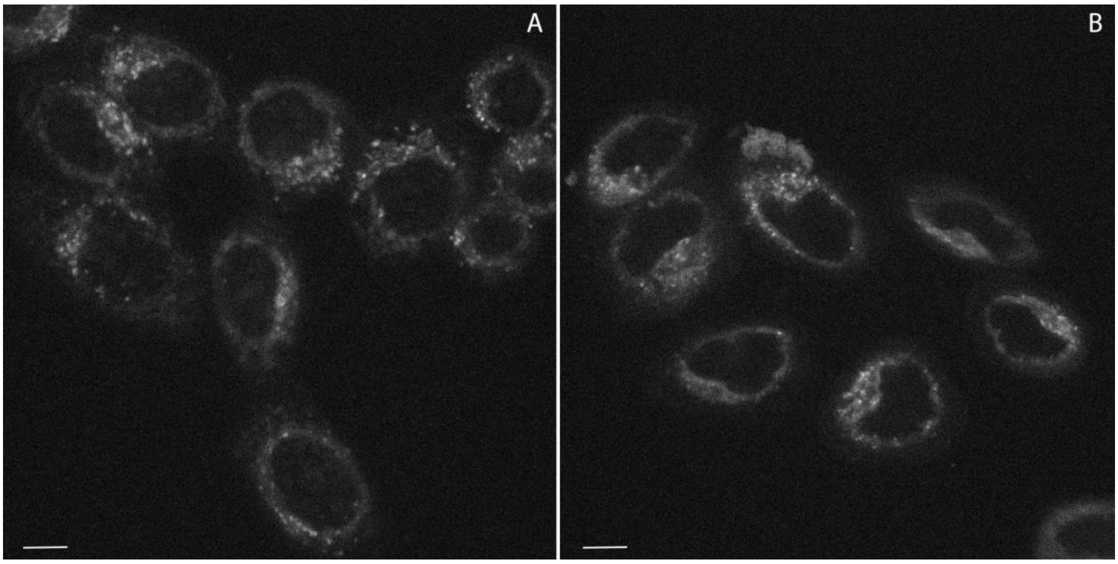

Fig. S4. A: confocal luminescence image of living HeLa cells stained with **1**. B: confocal luminescence image of living HeLa cells stained with **3**. Emission wavelengths are represented using a false colour. Scale bar 10 μm.

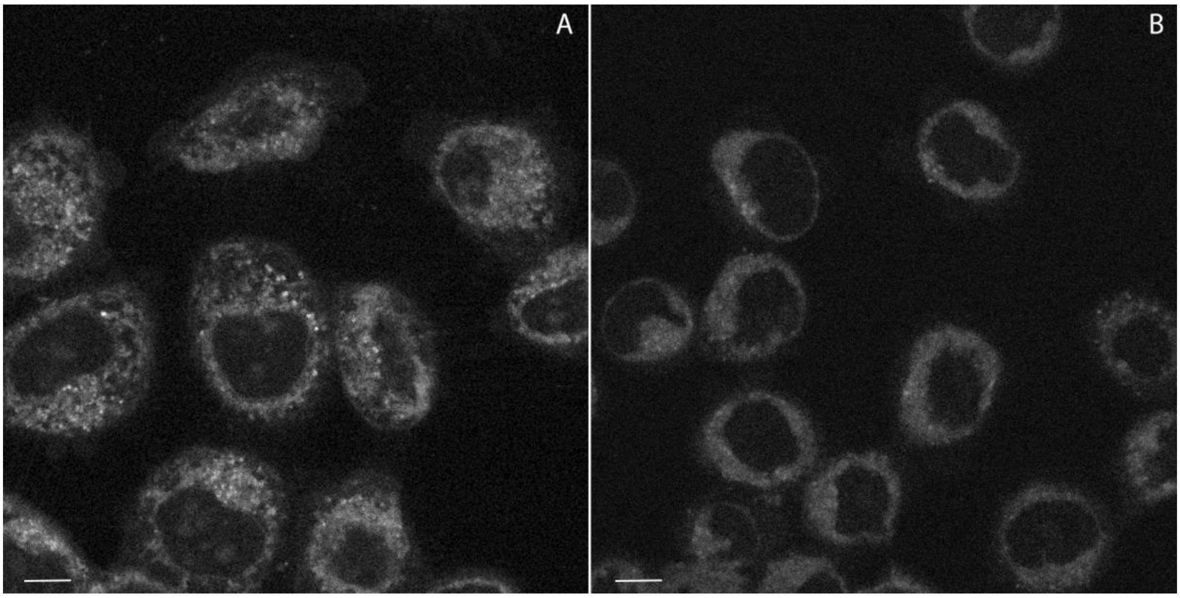

Fig. S5. A: confocal luminescence image of living HeLa cells stained with **1**. B: confocal luminescence image of living HeLa cells preincubated with FCCP and then stained with **1**. Emission wavelengths are represented using a false colour. Scale bar 10 μm.

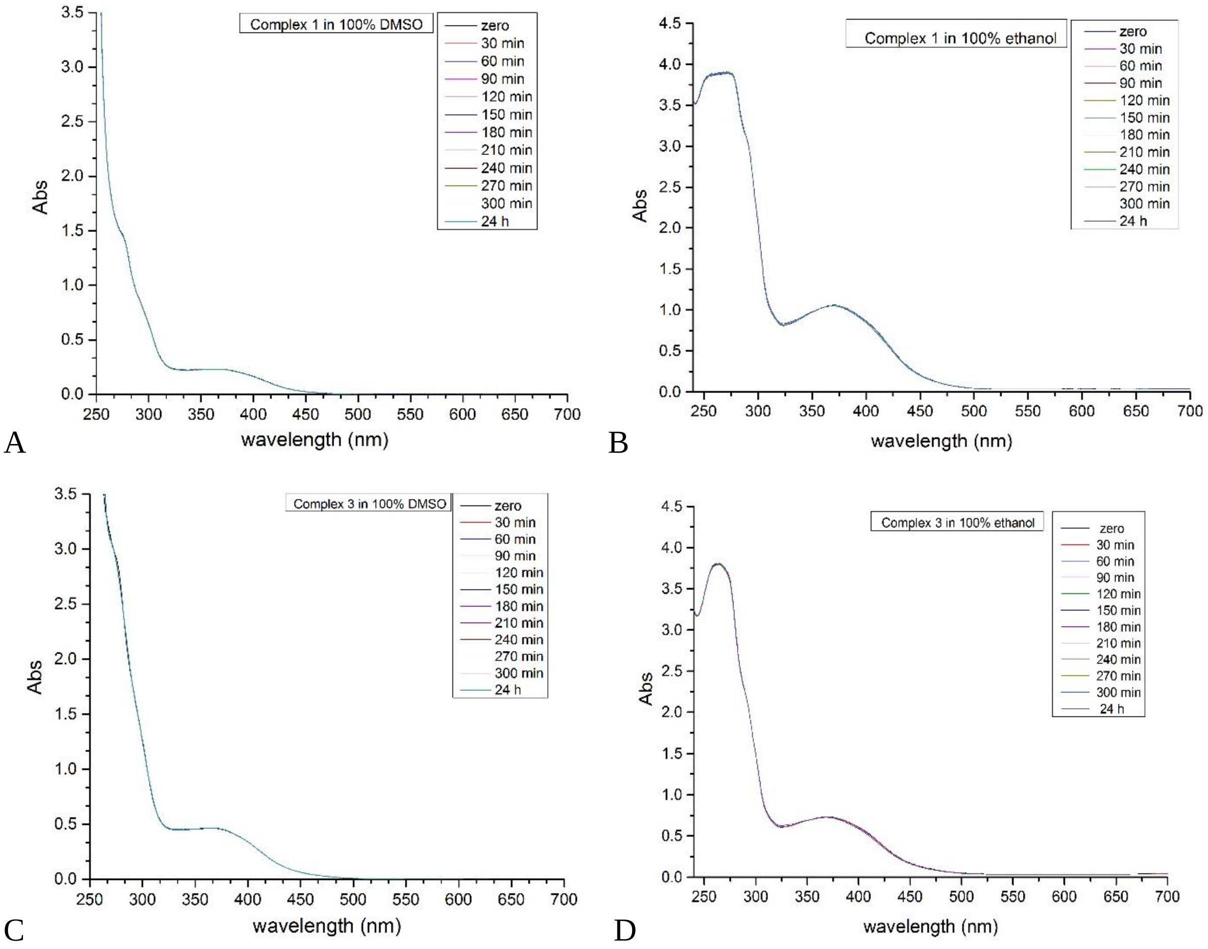

Fig. S6. UV-Vis spectra of 0.2 mM compound 1 or 3 in 100% DMSO (panels A-C) and 100% ethanol (panels B-D). Spectra were recorded for 24 h.

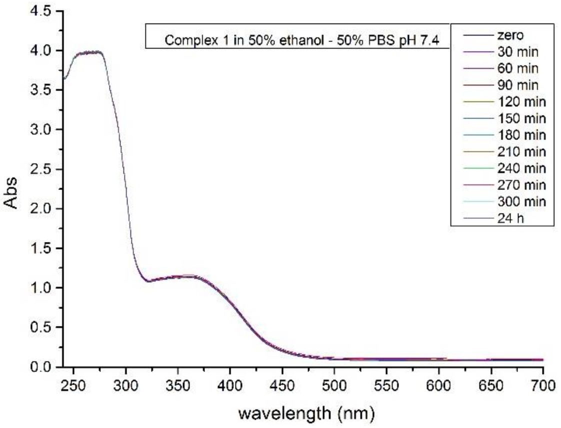
Complex 1 in 50% ethanol - 50% PBS pH 7.4
zero
30 min
60 min
90 min
120 min
150 min
180 min
210 min
240 min
270 min
300 min
24 h
Abs
wavelength (nm)

A

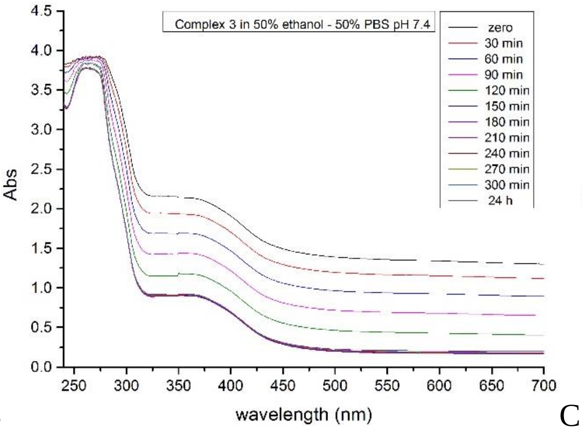
Complex 3 in 50% ethanol - 50% PBS pH 7.4
zero
30 min
60 min
90 min
120 min
150 min
180 min
210 min
240 min
270 min
300 min
24 h
Abs
wavelength (nm)

B

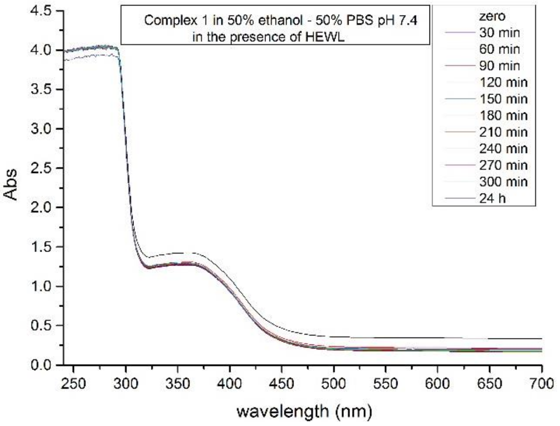
Complex 1 in 50% ethanol - 50% PBS pH 7.4
in the presence of HEWL
zero
30 min
60 min
90 min
120 min
150 min
180 min
210 min
240 min
270 min
300 min
24 h
Abs
wavelength (nm)

C

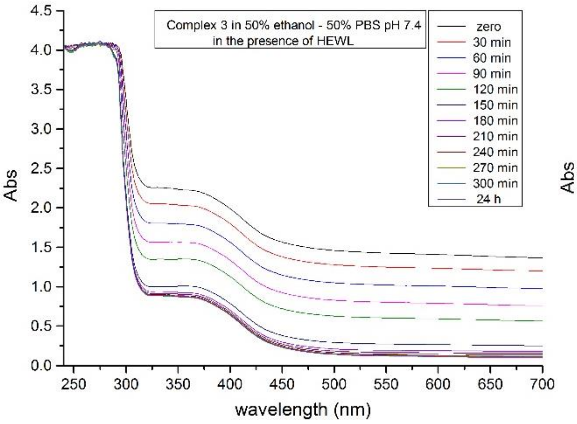
Complex 3 in 50% ethanol - 50% PBS pH 7.4
in the presence of HEWL
zero
30 min
60 min
90 min
120 min
150 min
180 min
210 min
240 min
270 min
300 min
24 h
Abs
wavelength (nm)

D

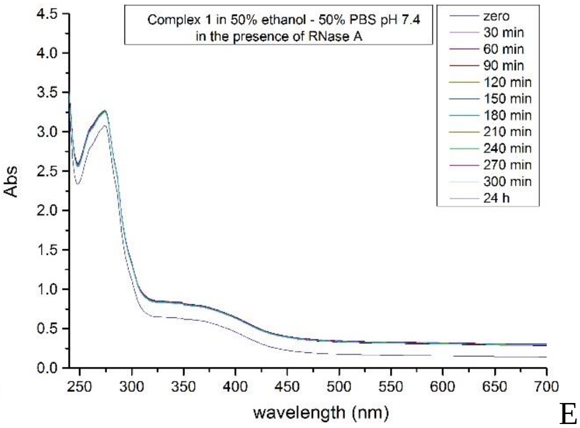
Complex 1 in 50% ethanol - 50% PBS pH 7.4
in the presence of RNase A
zero
30 min
60 min
90 min
120 min
150 min
180 min
210 min
240 min
270 min
300 min
24 h
Abs
wavelength (nm)

E

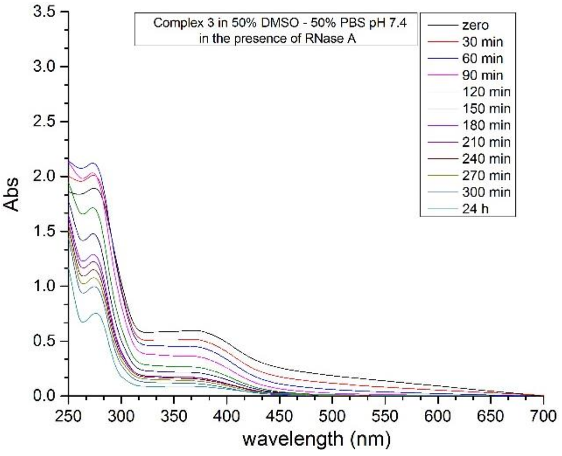
Complex 3 in 50% DMSO - 50% PBS pH 7.4
in the presence of RNase A
zero
30 min
60 min
90 min
120 min
150 min
180 min
210 min
240 min
270 min
300 min
24 h
Abs
wavelength (nm)

F

Fig. S7 UV-Vis spectra of 0.2 mM compound **1** or **3** in 50% ethanol and 50% PBS pH 7.4 in the absence (panels A-B) and in the presence of HEWL (panels C-D) and of RNase A (panels E-F) in a 1 : 3 protein to metal molar ratio. Spectra were recorded for 24 h. The change of the baseline for compound **3** in ethanol/PBS is probably due to the presence of molecules not completely dissolved in this mixture that scatters a fraction of light.

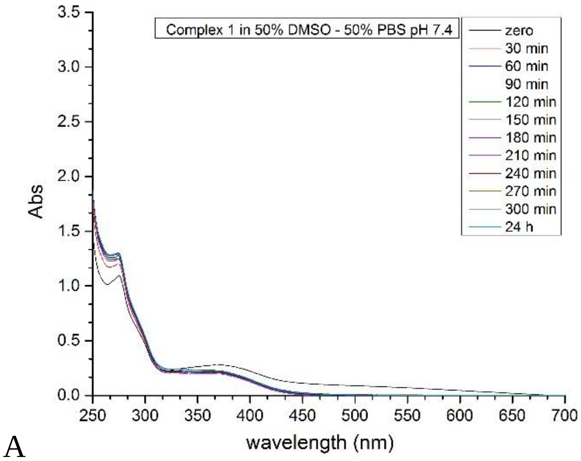
Complex 1 in 50% DMSO - 50% PBS pH 7.4
zero
30 min
60 min
90 min
120 min
150 min
180 min
210 min
240 min
270 min
300 min
24 h
Abs
wavelength (nm)

A

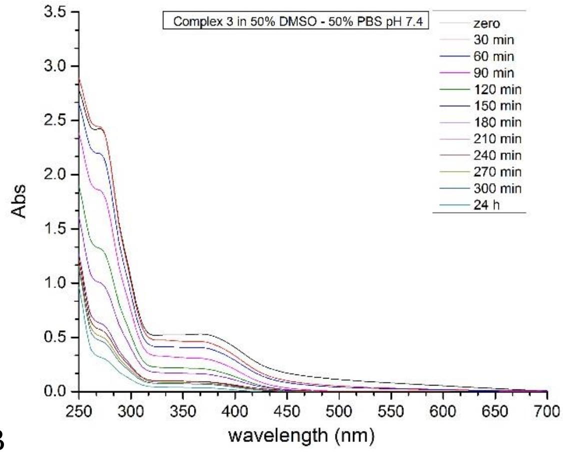
Complex 3 in 50% DMSO - 50% PBS pH 7.4
zero
30 min
60 min
90 min
120 min
150 min
180 min
210 min
240 min
270 min
300 min
24 h
Abs
wavelength (nm)

B

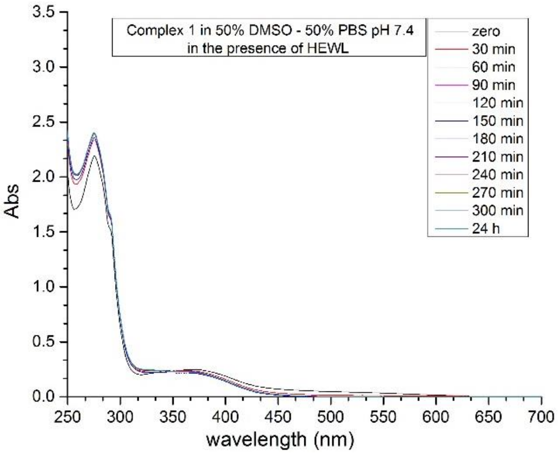
Complex 1 in 50% DMSO - 50% PBS pH 7.4
in the presence of HEWL
zero
30 min
60 min
90 min
120 min
150 min
180 min
210 min
240 min
270 min
300 min
24 h
Abs
wavelength (nm)

C

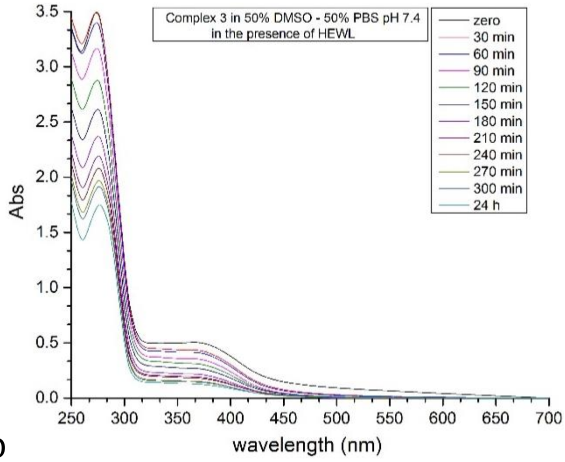
Complex 3 in 50% DMSO - 50% PBS pH 7.4
in the presence of HEWL
zero
30 min
60 min
90 min
120 min
150 min
180 min
210 min
240 min
270 min
300 min
24 h
Abs
wavelength (nm)

D

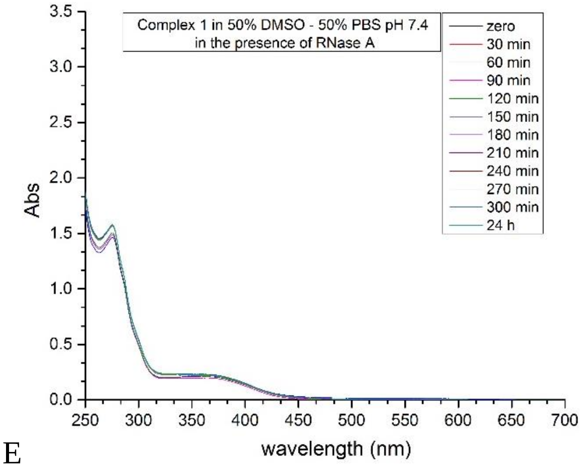
Complex 1 in 50% DMSO - 50% PBS pH 7.4
in the presence of RNase A
zero
30 min
60 min
90 min
120 min
150 min
180 min
210 min
240 min
270 min
300 min
24 h
Abs
wavelength (nm)

E

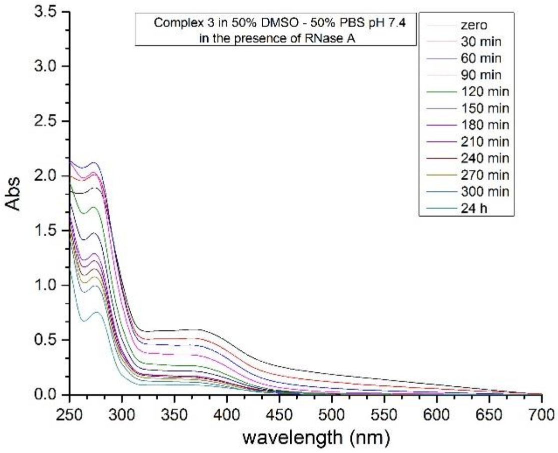
Complex 3 in 50% DMSO - 50% PBS pH 7.4
in the presence of RNase A
zero
30 min
60 min
90 min
120 min
150 min
180 min
210 min
240 min
270 min
300 min
24 h
Abs
wavelength (nm)

F

Fig. S8. UV-Vis spectra of 0.2 mM compound **1** or **3** in 50% DMSO and 50% PBS pH 7.4 in the absence (panels A-B) and in the presence of HEWL (panels C-D) and of RNase A (panels E-F) in a 1 : 3 protein to metal molar ratio. Spectra were recorded for 24 h.

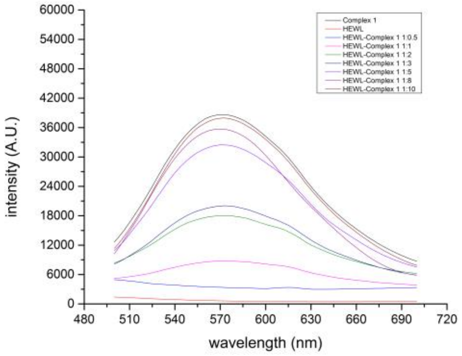

A

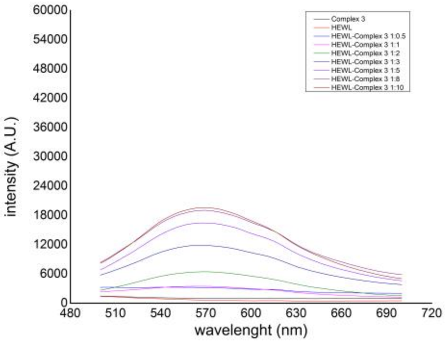

B

Fig. S9 Fluorescence spectra of HEWL (0.05 mg/mL in 10% ethanol) in the presence of increasing concentration of complex **1** (A) and complex **3** (B) upon excitation at 375nm.

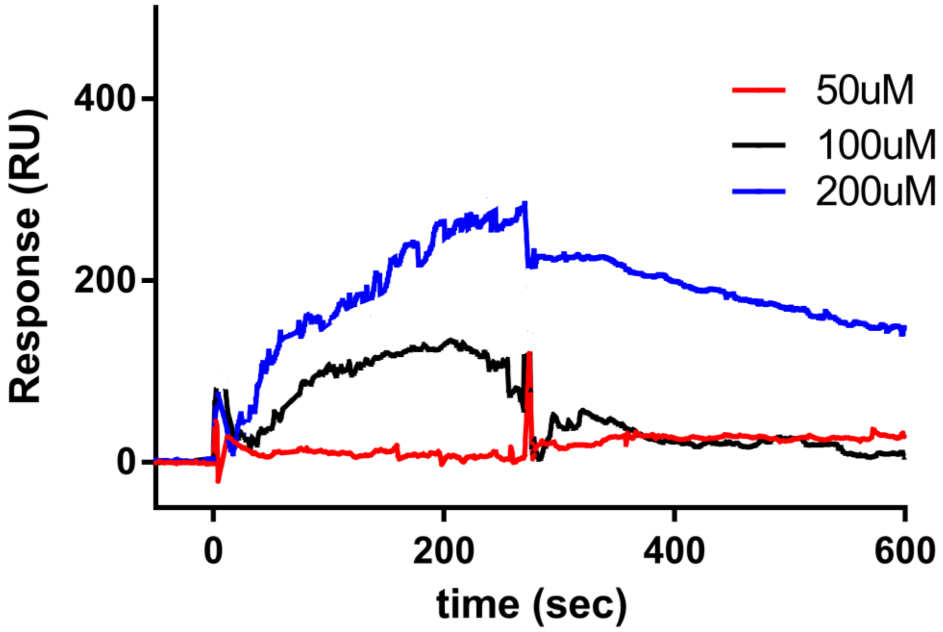

Fig. S10: Overlay of sensorgrams for the interaction between compound **3** and HEWL at 50, 100 and 200 μM.

Table S1 Crystallographic data and structural refinement details of **1–3**.

Compound	1	2	3
Empirical formula	$C_{22}H_{21}N_2O_5Re$	$C_{22}H_{17}N_2O_5Re$	$C_{58}H_{46}Fe_2N_4O_{10}Re_2$
Formula weight	579.61	575.58	1443.09
Temperature/K	100(2)	100(2)	100
Crystal system	Monoclinic	Monoclinic	Monoclinic
Space group	$P2_1/n$	$P2_1/n+$	$P2_1$
a/Å	6.32177(7)	6.19383(14)	10.61239(10)
b/Å	18.8683(2)	19.0776(5)	22.5597(2)
c/Å	17.3727(2)	17.0999(4)	10.88573(11)
β/°	94.4130(11)	98.541(2)	98.2886(10)
Volume/Å^3	2066.08(4)	1998.18(8)	2578.95(4)
Z	4	4	2
ρ_{calc}/g/cm^3	1.863	1.913	1.858
μ/mm^{-1}	5.918	6.119	5.295
F(000)	1128.0	1112.0	1408.0
Crystal size/mm^3	0.34 × 0.09 × 0.05	0.17 × 0.12 × 0.05	0.46 × 0.20 × 0.07
Radiation	Mo$K\alpha$ (λ = 0.71073 Å)	Mo$K\alpha$ (λ = 0.71073 Å)	Mo$K\alpha$ (λ = 0.71073 Å)
2Θ range for data collection/°	3.2 to 61.02	3.22 to 61.02	3.78 to 61.02
Index ranges	$-9 \le h \le 9$, $-26 \le k \le 26$, $-24 \le l \le 24$	$-8 \le h \le 8$, $-22 \le k \le 27$, $-24 \le l \le 24$	$-15 \le h \le 15$, $-32 \le k \le 32$, $-15 \le l \le 15$
Reflections collected	98524	19724of	125300
Independent reflections	6298 [R_{int} = 0.0529, R_{sigma} = 0.0172]	6097 [R_{int} = 0.0291, R_{sigma} = 0.0292]	15747 [R_{int} = 0.0719, R_{sigma} = 0.0338]
Data/restraints/parameters	6298/0/319	6097/0/271	15747/1/685
Goodness-of-fit on F^2	1.072	1.169	1.053
Final R indexes [I>=2σ (I)]	R_1 = 0.0160, wR_2 = 0.0392	R_1 = 0.0309, wR_2 = 0.0629	R_1 = 0.0237, wR_2 = 0.0564
Final R indexes [all data]	R_1 = 0.0176, wR_2 = 0.0399	R_1 = 0.0348, wR_2 = 0.0643	R_1 = 0.0244, wR_2 = 0.0571
Largest diff. peak/hole / e Å^{-3}	1.25/-0.97	3.67/-1.50	1.89/-0.91

Table S2. Bond lengths for **1**.

Atom	Atom	Length/Å	Atom	Atom	Length/Å
C1	O1	1.154(2)	C14	N1	1.363(2)
C1	Re1	1.922(2)	C15	N2	1.367(2)
C2	O2	1.155(2)	C16	C17A	1.511(6)
C2	Re1	1.9228(19)	C16	C17B	1.569(13)
C3	O3	1.164(2)	C16	O4	1.297(2)
C3	Re1	1.9002(17)	C16	O5	1.230(2)
C4	C5	1.402(3)	C17A	C18A	1.537(7)
C4	N1	1.337(2)	C17B	C18B	1.509(15)
C5	C6	1.373(3)	C18A	C19A	1.538(5)
C6	C7	1.413(3)	C18B	C19B	1.528(9)
C7	C8	1.433(3)	C19A	C20	1.446(4)
C7	C14	1.411(2)	C19B	C20	1.465(6)
C8	C9	1.354(3)	C20	C21A	1.498(4)
C9	C10	1.440(3)	C20	C21B	1.432(6)
C10	C11	1.411(3)	C21A	C22A	1.488(7)
C10	C15	1.401(2)	C21B	C22B	1.520(9)
C11	C12	1.374(3)	N1	Re1	2.1762(15)
C12	C13	1.402(3)	N2	Re1	2.1838(15)
C13	N2	1.334(2)	O4	Re1	2.1420(12)
C14	C15	1.435(2)			

Table S3 Valence angles for **1**.

Atom	Atom	Atom	Angle/°	Atom	Atom	Atom	Angle/°
O1	C1	Re1	177.23(18)	C20	C19A	C18A	118.2(3)
O2	C2	Re1	176.33(16)	C20	C19B	C18B	117.8(5)
O3	C3	Re1	178.18(15)	C19A	C20	C19B	67.1(3)
N1	C4	C5	122.72(18)	C19A	C20	C21A	119.3(3)
C6	C5	C4	119.87(17)	C19B	C20	C21A	152.0(3)
C5	C6	C7	119.04(18)	C21B	C20	C19A	152.7(3)
C6	C7	C8	123.37(17)	C21B	C20	C19B	123.1(4)
C14	C7	C6	117.49(18)	C21B	C20	C21A	39.0(3)
C14	C7	C8	119.12(17)	C22A	C21A	C20	117.2(4)
C9	C8	C7	121.03(17)	C20	C21B	C22B	120.6(5)
C8	C9	C10	120.99(18)	C4	N1	C14	117.85(16)
C11	C10	C9	123.35(17)	C4	N1	Re1	127.14(13)
C15	C10	C9	119.16(16)	C14	N1	Re1	115.00(11)
C15	C10	C11	117.49(16)	C13	N2	C15	118.19(15)
C12	C11	C10	119.25(17)	C13	N2	Re1	127.12(12)
C11	C12	C13	119.80(16)	C15	N2	Re1	114.63(11)
N2	C13	C12	122.31(16)	C16	O4	Re1	124.31(11)
C7	C14	C15	119.73(16)	C1	Re1	C2	89.24(8)
N1	C14	C7	123.03(16)	C1	Re1	N1	96.42(7)
N1	C14	C15	117.24(14)	C1	Re1	N2	171.79(7)
C10	C15	C14	119.91(15)	C1	Re1	O4	95.58(7)
N2	C15	C10	122.96(15)	C2	Re1	N1	174.23(7)
N2	C15	C14	117.13(15)	C2	Re1	N2	98.36(7)
C17A	C16	C17B	17.1(3)	C2	Re1	O4	96.98(6)
O4	C16	C17A	113.8(3)	C3	Re1	C1	87.35(8)
O4	C16	C17B	109.6(5)	C3	Re1	C2	87.14(7)
O5	C16	C17A	120.5(3)	C3	Re1	N1	94.19(6)
O5	C16	C17B	124.2(5)	C3	Re1	N2	96.08(6)
O5	C16	O4	125.33(16)	C3	Re1	O4	174.96(6)
C16	C17A	C18A	116.2(4)	N1	Re1	N2	75.93(5)
C18B	C17B	C16	116.9(9)	O4	Re1	N1	81.42(5)
C17A	C18A	C19A	111.8(3)	O4	Re1	N2	80.48(5)
C17B	C18B	C19B	111.5(7)				

Table S4 Torsion angles for **1**.

A	B	C	D	Angle/°	A	B	C	D	Angle/°
C4	C5	C6	C7	-0.2(3)	C15	N2	Re1	C3	-95.00(12)
C4	N1	Re1	C1	4.88(15)	C15	N2	Re1	N1	-2.18(11)
C4	N1	Re1	C2	173.9(6)	C15	N2	Re1	O4	81.28(11)
C4	N1	Re1	C3	-82.92(15)	C16	C17A	C18A	C19A	-164.9(3)
C4	N1	Re1	N2	-178.14(15)	C16	C17B	C18B	C19B	173.8(6)
C4	N1	Re1	O4	99.59(14)	C16	O4	Re1	C1	-115.24(15)
C5	C4	N1	C14	-0.6(2)	C16	O4	Re1	C2	-25.32(15)
C5	C4	N1	Re1	178.63(13)	C16	O4	Re1	C3	119.3(7)
C5	C6	C7	C8	178.04(18)	C16	O4	Re1	N1	149.09(15)
C5	C6	C7	C14	-0.4(3)	C16	O4	Re1	N2	72.04(14)
C6	C7	C8	C9	-179.45(19)	C17A	C16	C17B	C18B	91(2)
C6	C7	C14	C15	-178.95(16)	C17A	C16	O4	Re1	-164.0(2)
C6	C7	C14	N1	0.6(3)	C17A	C18A	C19A	C20	-60.7(4)
C7	C8	C9	C10	-1.0(3)	C17B	C16	C17A	C18A	-88(2)
C7	C14	C15	C10	-2.1(2)	C17B	C16	O4	Re1	178.1(4)
C7	C14	C15	N2	177.42(15)	C17B	C18B	C19B	C20	60.5(8)
C7	C14	N1	C4	-0.1(2)	C18A	C19A	C20	C19B	-38.7(3)
C7	C14	N1	Re1	-179.41(13)	C18A	C19A	C20	C21A	171.8(3)
C8	C7	C14	C15	2.5(3)	C18A	C19A	C20	C21B	-157.0(6)
C8	C7	C14	N1	-177.92(16)	C18B	C19B	C20	C19A	38.4(5)
C8	C9	C10	C11	-178.15(19)	C18B	C19B	C20	C21A	148.2(7)
C8	C9	C10	C15	1.4(3)	C18B	C19B	C20	C21B	-170.4(5)
C9	C10	C11	C12	179.16(18)	C19A	C20	C21A	C22A	-179.5(3)
C9	C10	C15	C14	0.2(2)	C19A	C20	C21B	C22B	-59.9(9)
C9	C10	C15	N2	-179.34(16)	C19B	C20	C21A	C22A	84.4(8)
C10	C11	C12	C13	0.3(3)	C19B	C20	C21B	C22B	-164.4(5)
C10	C15	N2	C13	0.1(2)	C21A	C20	C21B	C22B	-14.0(4)
C10	C15	N2	Re1	-177.46(13)	C21B	C20	C21A	C22A	22.7(5)
C11	C10	C15	C14	179.72(16)	N1	C4	C5	C6	0.7(3)
C11	C10	C15	N2	0.2(3)	N1	C14	C15	C10	178.32(15)
C11	C12	C13	N2	0.0(3)	N1	C14	C15	N2	-2.2(2)
C12	C13	N2	C15	-0.2(2)	O1	C1	Re1	C2	132(4)
C12	C13	N2	Re1	176.99(12)	O1	C1	Re1	C3	45(4)
C13	N2	Re1	C1	-157.9(4)	O1	C1	Re1	N1	-49(4)
C13	N2	Re1	C2	-0.25(15)	O1	C1	Re1	N2	-70(4)
C13	N2	Re1	C3	87.74(15)	O1	C1	Re1	O4	-131(4)
C13	N2	Re1	N1	-179.44(15)	O2	C2	Re1	C1	-77(3)
C13	N2	Re1	O4	-95.98(14)	O2	C2	Re1	C3	11(3)
C14	C7	C8	C9	-1.0(3)	O2	C2	Re1	N1	114(3)
C14	C15	N2	C13	-179.45(15)	O2	C2	Re1	N2	106(3)

C14	C15	N2	Re1	3.03(18)	O2	C2	Re1	O4	-172(3)
C14	N1	Re1	C1	-175.91(12)	O3	C3	Re1	C1	70(5)
C14	N1	Re1	C2	-6.9(7)	O3	C3	Re1	C2	-19(5)
C14	N1	Re1	C3	96.29(12)	O3	C3	Re1	N1	167(5)
C14	N1	Re1	N2	1.06(11)	O3	C3	Re1	N2	-117(5)
C14	N1	Re1	O4	-81.20(12)	O3	C3	Re1	O4	-164(4)
C15	C10	C11	C12	-0.4(3)	O4	C16	C17A	C18A	-167.0(3)
C15	C14	N1	C4	179.44(15)	O4	C16	C17B	C18B	-161.4(6)
C15	C14	N1	Re1	0.15(18)	O5	C16	C17A	C18A	20.1(5)
C15	N2	Re1	C1	19.4(5)	O5	C16	C17B	C18B	8.4(9)
C15	N2	Re1	C2	177.01(12)	O5	C16	O4	Re1	8.4(3)

Table S5 Bond lengths for **2**.

Atom	Atom	Length/Å	Atom	Atom	Length/Å
C1	O1	1.151(5)	C12	C13	1.402(5)
C1	Re1	1.924(4)	C13	N2	1.333(4)
C2	O2	1.152(4)	C14	C15	1.430(4)
C2	Re1	1.926(3)	C14	N1	1.366(4)
C3	O3	1.165(4)	C15	N2	1.370(4)
C3	Re1	1.903(3)	C16	C17	1.512(5)
C4	C5	1.401(5)	C16	O4	1.295(4)
C4	N1	1.334(4)	C16	O5	1.234(4)
C5	C6	1.373(5)	C17	C18	1.528(5)
C6	C7	1.409(5)	C18	C19	1.515(5)
C7	C8	1.433(5)	C19	C20	1.530(6)
C7	C14	1.405(4)	C20	C21	1.471(6)
C8	C9	1.356(5)	C21	C22	1.174(6)
C9	C10	1.438(5)	N1	Re1	2.173(3)
C10	C11	1.406(5)	N2	Re1	2.185(3)
C10	C15	1.403(5)	O4	Re1	2.146(2)
C11	C12	1.374(5)			

Table S6 Valence angles for **2**.

Atom	Atom	Atom	Angle/°	Atom	Atom	Atom	Angle/°
O1	C1	Re1	177.8(3)	C19	C18	C17	112.2(3)
O2	C2	Re1	176.7(3)	C18	C19	C20	111.4(4)
O3	C3	Re1	178.2(3)	C21	C20	C19	112.4(4)
N1	C4	C5	122.6(3)	C22	C21	C20	177.8(6)
C6	C5	C4	119.7(3)	C4	N1	C14	118.1(3)
C5	C6	C7	119.1(3)	C4	N1	Re1	126.9(2)
C6	C7	C8	123.3(3)	C14	N1	Re1	115.0(2)
C14	C7	C6	117.7(3)	C13	N2	C15	118.1(3)
C14	C7	C8	119.0(3)	C13	N2	Re1	127.2(2)
C9	C8	C7	120.8(3)	C15	N2	Re1	114.4(2)
C8	C9	C10	121.3(3)	C16	O4	Re1	122.2(2)
C11	C10	C9	123.7(3)	C1	Re1	C2	90.05(15)
C15	C10	C9	118.7(3)	C1	Re1	N1	97.26(13)
C15	C10	C11	117.6(3)	C1	Re1	N2	171.41(13)
C12	C11	C10	119.4(3)	C1	Re1	O4	94.89(13)
C11	C12	C13	119.6(3)	C2	Re1	N1	172.51(13)
N2	C13	C12	122.5(3)	C2	Re1	N2	96.67(13)
C7	C14	C15	120.0(3)	C2	Re1	O4	97.00(12)
N1	C14	C7	122.8(3)	C3	Re1	C1	86.68(15)
N1	C14	C15	117.2(3)	C3	Re1	C2	88.05(15)
C10	C15	C14	120.1(3)	C3	Re1	N1	93.96(12)
N2	C15	C10	122.8(3)	C3	Re1	N2	98.87(12)
N2	C15	C14	117.1(3)	C3	Re1	O4	174.70(12)
O4	C16	C17	115.1(3)	N1	Re1	N2	75.90(10)
O5	C16	C17	119.7(3)	O4	Re1	N1	80.83(9)
O5	C16	O4	125.0(3)	O4	Re1	N2	79.01(10)
C16	C17	C18	108.8(3)				

Table S7 Torsion angles for **2**.

A	B	C	D	Angle/°	A	B	C	D	Angle/°
C4	C5	C6	C7	-0.4(5)	C14	N1	Re1	C3	101.9(2)
C4	N1	Re1	C1	8.6(3)	C14	N1	Re1	N2	3.7(2)
C4	N1	Re1	C2	176.1(9)	C14	N1	Re1	O4	-77.2(2)
C4	N1	Re1	C3	-78.6(3)	C15	C10	C11	C12	-0.9(5)
C4	N1	Re1	N2	-176.7(3)	C15	C14	N1	C4	178.5(3)
C4	N1	Re1	O4	102.4(3)	C15	C14	N1	Re1	-1.9(4)
C5	C4	N1	C14	0.3(5)	C15	N2	Re1	C1	32.7(9)
C5	C4	N1	Re1	-179.3(2)	C15	N2	Re1	C2	173.9(2)
C5	C6	C7	C8	177.9(3)	C15	N2	Re1	C3	-97.0(2)
C5	C6	C7	C14	-0.8(5)	C15	N2	Re1	N1	-5.2(2)
C6	C7	C8	C9	179.8(3)	C15	N2	Re1	O4	78.0(2)
C6	C7	C14	C15	-178.3(3)	C16	C17	C18	C19	-176.2(3)
C6	C7	C14	N1	1.8(5)	C16	O4	Re1	C1	-116.4(3)
C7	C8	C9	C10	-0.5(5)	C16	O4	Re1	C2	-25.7(3)
C7	C14	C15	C10	-2.4(5)	C16	O4	Re1	C3	136.6(12)
C7	C14	C15	N2	177.2(3)	C16	O4	Re1	N1	147.0(3)
C7	C14	N1	C4	-1.5(5)	C16	O4	Re1	N2	69.7(3)
C7	C14	N1	Re1	178.1(2)	C17	C16	O4	Re1	-165.8(2)
C8	C7	C14	C15	2.9(5)	C17	C18	C19	C20	179.8(3)
C8	C7	C14	N1	-177.0(3)	C18	C19	C20	C21	-66.1(5)
C8	C9	C10	C11	-179.0(3)	C19	C20	C21	C22	-36(14)
C8	C9	C10	C15	1.0(5)	N1	C4	C5	C6	0.6(5)
C9	C10	C11	C12	179.1(3)	N1	C14	C15	C10	177.5(3)
C9	C10	C15	C14	0.5(5)	N1	C14	C15	N2	-2.8(4)
C9	C10	C15	N2	-179.2(3)	O1	C1	Re1	C2	131(9)
C10	C11	C12	C13	0.6(5)	O1	C1	Re1	C3	43(9)
C10	C15	N2	C13	-0.4(5)	O1	C1	Re1	N1	-51(9)
C10	C15	N2	Re1	-174.3(2)	O1	C1	Re1	N2	-88(9)
C11	C10	C15	C14	-179.5(3)	O1	C1	Re1	O4	-132(9)
C11	C10	C15	N2	0.8(5)	O2	C2	Re1	C1	-105(6)
C11	C12	C13	N2	-0.1(5)	O2	C2	Re1	C3	-19(6)
C12	C13	N2	C15	0.0(5)	O2	C2	Re1	N1	87(6)
C12	C13	N2	Re1	173.1(2)	O2	C2	Re1	N2	80(6)
C13	N2	Re1	C1	-140.6(8)	O2	C2	Re1	O4	160(6)
C13	N2	Re1	C2	0.6(3)	O3	C3	Re1	C1	3(10)
C13	N2	Re1	C3	89.6(3)	O3	C3	Re1	C2	-87(10)
C13	N2	Re1	N1	-178.5(3)	O3	C3	Re1	N1	101(10)
C13	N2	Re1	O4	-95.3(3)	O3	C3	Re1	N2	177(100)

C14	C7	C8	C9	-1.5(5)	O3	C3	Re1	O4	111(10)
C14	C15	N2	C13	180.0(3)	O4	C16	C17	C18	97.6(4)
C14	C15	N2	Re1	6.0(4)	O5	C16	C17	C18	-78.5(4)
C14	N1	Re1	C1	-171.0(2)	O5	C16	O4	Re1	10.1(5)
C14	N1	Re1	C2	-3.5(11)					

Table S8 Bond lengths for **3**.

Atom	Atom	Length/Å	Atom	Atom	Length/Å
C1A	O1A	1.164(4)	C1B	O1B	1.174(5)
C1A	Re1A	1.903(4)	C1B	Re1B	1.901(4)
C2A	O2A	1.160(4)	C2B	O2B	1.167(5)
C2A	Re1A	1.917(3)	C2B	Re1B	1.911(4)
C3A	O3A	1.157(4)	C3B	O3B	1.152(4)
C3A	Re1A	1.910(3)	C3B	Re1B	1.919(4)
C4A	C5A	1.401(5)	C4B	C5B	1.396(5)
C4A	N1A	1.332(4)	C4B	N1B	1.331(4)
C5A	C6A	1.372(5)	C5B	C6B	1.369(5)
C6A	C7A	1.405(4)	C6B	C7B	1.411(4)
C7A	C8A	1.426(5)	C7B	C8B	1.435(4)
C7A	C14A	1.411(4)	C7B	C14B	1.404(4)
C8A	C9A	1.360(5)	C8B	C9B	1.353(5)
C9A	C10A	1.435(4)	C9B	C10B	1.431(5)
C10A	C11A	1.420(4)	C10B	C11B	1.403(5)
C10A	C15A	1.402(4)	C10B	C15B	1.409(4)
C11A	C12A	1.372(5)	C11B	C12B	1.369(5)
C12A	C13A	1.398(4)	C12B	C13B	1.408(5)
C13A	N2A	1.341(4)	C13B	N2B	1.342(4)
C14A	C15A	1.432(4)	C14B	C15B	1.434(4)
C14A	N1A	1.363(4)	C14B	N1B	1.357(4)
C15A	N2A	1.361(4)	C15B	N2B	1.368(4)
C16A	C17A	1.529(4)	C16B	C17B	1.529(5)
C16A	O4A	1.301(4)	C16B	O4B	1.286(4)
C16A	O5A	1.224(4)	C16B	O5B	1.234(4)
C17A	C18A	1.534(5)	C17B	C18B	1.528(5)
C18A	C19A	1.531(4)	C18B	C19B	1.528(4)
C19A	C20A	1.507(5)	C19B	C20B	1.510(4)
C20A	C21A	1.423(5)	C20B	C21B	1.431(4)
C20A	C24A	1.424(4)	C20B	C24B	1.429(4)
C20A	Fe1A	2.060(3)	C20B	Fe1B	2.058(3)
C21A	C22A	1.435(5)	C21B	C22B	1.425(5)
C21A	Fe1A	2.043(3)	C21B	Fe1B	2.040(4)
C22A	C23A	1.420(5)	C22B	C23B	1.435(5)
C22A	Fe1A	2.047(3)	C22B	Fe1B	2.048(3)
C23A	C24A	1.436(5)	C23B	C24B	1.414(5)
C23A	Fe1A	2.055(3)	C23B	Fe1B	2.046(3)
C24A	Fe1A	2.058(3)	C24B	Fe1B	2.046(3)
C25A	C26A	1.426(5)	C25B	C26B	1.421(5)
C25A	C29A	1.429(5)	C25B	C29B	1.432(5)

C25A	Fe1A	2.047(3)	C25B	Fe1B	2.036(3)
C26A	C27A	1.424(5)	C26B	C27B	1.418(5)
C26A	Fe1A	2.052(3)	C26B	Fe1B	2.044(3)
C27A	C28A	1.426(5)	C27B	C28B	1.427(5)
C27A	Fe1A	2.048(3)	C27B	Fe1B	2.052(4)
C28A	C29A	1.420(5)	C28B	C29B	1.425(5)
C28A	Fe1A	2.051(3)	C28B	Fe1B	2.058(4)
C29A	Fe1A	2.043(3)	C29B	Fe1B	2.052(3)
N1A	Re1A	2.177(3)	N1B	Re1B	2.181(3)
N2A	Re1A	2.175(3)	N2B	Re1B	2.180(3)
O4A	Re1A	2.125(2)	O4B	Re1B	2.129(2)

Table S9 Valence angles for **3**.

Atom	Atom	Atom	Angle/°	Atom	Atom	Atom	Angle/°
O1A	C1A	Re1A	179.1(3)	O1B	C1B	Re1B	174.4(4)
O2A	C2A	Re1A	175.4(3)	O2B	C2B	Re1B	178.2(3)
O3A	C3A	Re1A	177.9(3)	O3B	C3B	Re1B	176.1(3)
N1A	C4A	C5A	122.1(3)	N1B	C4B	C5B	123.5(3)
C6A	C5A	C4A	119.8(3)	C6B	C5B	C4B	119.1(3)
C5A	C6A	C7A	119.5(3)	C5B	C6B	C7B	119.3(3)
C6A	C7A	C8A	123.8(3)	C6B	C7B	C8B	123.5(3)
C6A	C7A	C14A	117.5(3)	C14B	C7B	C6B	117.6(3)
C14A	C7A	C8A	118.8(3)	C14B	C7B	C8B	118.9(3)
C9A	C8A	C7A	121.2(3)	C9B	C8B	C7B	121.4(3)
C8A	C9A	C10A	120.9(3)	C8B	C9B	C10B	120.8(3)
C11A	C10A	C9A	123.8(3)	C11B	C10B	C9B	123.4(3)
C15A	C10A	C9A	119.1(3)	C11B	C10B	C15B	117.5(3)
C15A	C10A	C11A	117.1(3)	C15B	C10B	C9B	119.2(3)
C12A	C11A	C10A	119.1(3)	C12B	C11B	C10B	120.0(3)
C11A	C12A	C13A	120.0(3)	C11B	C12B	C13B	119.2(3)
N2A	C13A	C12A	122.3(3)	N2B	C13B	C12B	122.7(3)
C7A	C14A	C15A	120.2(3)	C7B	C14B	C15B	119.8(3)
N1A	C14A	C7A	122.4(3)	N1B	C14B	C7B	122.9(3)
N1A	C14A	C15A	117.4(3)	N1B	C14B	C15B	117.3(3)
C10A	C15A	C14A	119.8(3)	C10B	C15B	C14B	119.8(3)
N2A	C15A	C10A	123.4(3)	N2B	C15B	C10B	122.8(3)
N2A	C15A	C14A	116.8(3)	N2B	C15B	C14B	117.3(3)
O4A	C16A	C17A	112.7(3)	O4B	C16B	C17B	114.2(3)
O5A	C16A	C17A	121.6(3)	O5B	C16B	C17B	120.2(3)
O5A	C16A	O4A	125.7(3)	O5B	C16B	O4B	125.5(3)
C16A	C17A	C18A	111.7(3)	C18B	C17B	C16B	114.9(3)
C19A	C18A	C17A	110.8(3)	C17B	C18B	C19B	112.2(3)
C20A	C19A	C18A	115.0(3)	C20B	C19B	C18B	114.2(3)
C19A	C20A	Fe1A	128.3(2)	C19B	C20B	Fe1B	128.9(2)
C21A	C20A	C19A	129.6(3)	C21B	C20B	C19B	128.3(3)
C21A	C20A	C24A	107.4(3)	C21B	C20B	Fe1B	68.87(19)
C21A	C20A	Fe1A	69.09(19)	C24B	C20B	C19B	125.1(3)
C24A	C20A	C19A	123.0(3)	C24B	C20B	C21B	106.6(3)
C24A	C20A	Fe1A	69.68(18)	C24B	C20B	Fe1B	69.18(19)
C20A	C21A	C22A	108.8(3)	C20B	C21B	Fe1B	70.26(19)
C20A	C21A	Fe1A	70.33(18)	C22B	C21B	C20B	109.0(3)
C22A	C21A	Fe1A	69.57(18)	C22B	C21B	Fe1B	69.9(2)
C21A	C22A	Fe1A	69.34(19)	C21B	C22B	C23B	107.4(3)
C23A	C22A	C21A	107.5(3)	C21B	C22B	Fe1B	69.28(19)

C23A	C22A	Fe1A	70.07(18)	C23B	C22B	Fe1B	69.42(19)
C22A	C23A	C24A	108.0(3)	C22B	C23B	Fe1B	69.53(19)
C22A	C23A	Fe1A	69.41(18)	C24B	C23B	C22B	107.8(3)
C24A	C23A	Fe1A	69.64(18)	C24B	C23B	Fe1B	69.78(18)
C20A	C24A	C23A	108.4(3)	C20B	C24B	Fe1B	70.07(19)
C20A	C24A	Fe1A	69.85(18)	C23B	C24B	C20B	109.2(3)
C23A	C24A	Fe1A	69.47(18)	C23B	C24B	Fe1B	69.78(19)
C26A	C25A	C29A	107.9(3)	C26B	C25B	C29B	107.9(3)
C26A	C25A	Fe1A	69.83(19)	C26B	C25B	Fe1B	69.93(19)
C29A	C25A	Fe1A	69.42(18)	C29B	C25B	Fe1B	70.10(19)
C25A	C26A	Fe1A	69.45(18)	C25B	C26B	Fe1B	69.31(19)
C27A	C26A	C25A	108.0(3)	C27B	C26B	C25B	108.5(3)
C27A	C26A	Fe1A	69.54(18)	C27B	C26B	Fe1B	70.0(2)
C26A	C27A	C28A	107.9(3)	C26B	C27B	C28B	107.8(3)
C26A	C27A	Fe1A	69.81(19)	C26B	C27B	Fe1B	69.4(2)
C28A	C27A	Fe1A	69.75(19)	C28B	C27B	Fe1B	69.9(2)
C27A	C28A	Fe1A	69.54(19)	C27B	C28B	Fe1B	69.5(2)
C29A	C28A	C27A	108.3(3)	C29B	C28B	C27B	108.2(3)
C29A	C28A	Fe1A	69.42(18)	C29B	C28B	Fe1B	69.50(19)
C25A	C29A	Fe1A	69.67(18)	C25B	C29B	Fe1B	68.88(19)
C28A	C29A	C25A	107.9(3)	C28B	C29B	C25B	107.6(3)
C28A	C29A	Fe1A	70.00(19)	C28B	C29B	Fe1B	69.9(2)
C21A	Fe1A	C20A	40.58(13)	C21B	Fe1B	C20B	40.87(13)
C21A	Fe1A	C22A	41.09(14)	C21B	Fe1B	C22B	40.81(13)
C21A	Fe1A	C23A	68.36(13)	C21B	Fe1B	C23B	68.68(14)
C21A	Fe1A	C24A	68.05(13)	C21B	Fe1B	C24B	68.29(14)
C21A	Fe1A	C25A	119.73(14)	C21B	Fe1B	C26B	106.70(14)
C21A	Fe1A	C26A	107.30(13)	C21B	Fe1B	C27B	126.01(14)
C21A	Fe1A	C27A	125.45(14)	C21B	Fe1B	C28B	164.30(14)
C21A	Fe1A	C28A	163.05(15)	C21B	Fe1B	C29B	153.09(14)
C22A	Fe1A	C20A	68.92(14)	C22B	Fe1B	C20B	68.95(14)
C22A	Fe1A	C23A	40.51(13)	C22B	Fe1B	C27B	107.19(15)
C22A	Fe1A	C24A	68.53(14)	C22B	Fe1B	C28B	127.18(15)
C22A	Fe1A	C25A	155.23(14)	C22B	Fe1B	C29B	165.22(14)
C22A	Fe1A	C26A	120.49(14)	C23B	Fe1B	C20B	68.78(13)
C22A	Fe1A	C27A	107.77(14)	C23B	Fe1B	C22B	41.04(14)
C22A	Fe1A	C28A	125.76(15)	C23B	Fe1B	C24B	40.44(13)
C23A	Fe1A	C20A	68.62(13)	C23B	Fe1B	C27B	119.49(14)
C23A	Fe1A	C24A	40.88(13)	C23B	Fe1B	C28B	108.74(14)
C24A	Fe1A	C20A	40.47(12)	C23B	Fe1B	C29B	127.60(13)
C25A	Fe1A	C20A	106.61(13)	C24B	Fe1B	C20B	40.75(12)
C25A	Fe1A	C23A	162.49(14)	C24B	Fe1B	C22B	68.45(14)
C25A	Fe1A	C24A	124.76(14)	C24B	Fe1B	C27B	154.20(14)
C25A	Fe1A	C26A	40.72(14)	C24B	Fe1B	C28B	120.51(13)
C25A	Fe1A	C27A	68.56(14)	C24B	Fe1B	C29B	108.85(13)
C25A	Fe1A	C28A	68.41(14)	C25B	Fe1B	C20B	106.91(13)
C26A	Fe1A	C20A	124.56(13)	C25B	Fe1B	C21B	117.98(14)
C26A	Fe1A	C23A	155.78(14)	C25B	Fe1B	C22B	152.15(14)

C26A	Fe1A	C24A	161.64(14)	C25B	Fe1B	C23B	165.21(14)
C27A	Fe1A	C20A	161.99(13)	C25B	Fe1B	C24B	127.32(13)
C27A	Fe1A	C23A	121.13(14)	C25B	Fe1B	C26B	40.77(14)
C27A	Fe1A	C24A	156.39(14)	C25B	Fe1B	C27B	68.61(14)
C27A	Fe1A	C26A	40.65(14)	C25B	Fe1B	C28B	68.55(13)
C27A	Fe1A	C28A	40.71(13)	C25B	Fe1B	C29B	41.02(13)
C28A	Fe1A	C20A	155.39(14)	C26B	Fe1B	C20B	126.01(14)
C28A	Fe1A	C23A	108.49(13)	C26B	Fe1B	C22B	118.06(15)
C28A	Fe1A	C24A	121.26(13)	C26B	Fe1B	C23B	153.11(14)
C28A	Fe1A	C26A	68.33(13)	C26B	Fe1B	C24B	164.49(14)
C29A	Fe1A	C20A	119.87(13)	C26B	Fe1B	C27B	40.51(14)
C29A	Fe1A	C21A	154.80(14)	C26B	Fe1B	C28B	68.18(14)
C29A	Fe1A	C22A	162.62(14)	C26B	Fe1B	C29B	68.55(14)
C29A	Fe1A	C23A	125.60(13)	C27B	Fe1B	C20B	163.59(13)
C29A	Fe1A	C24A	107.56(13)	C27B	Fe1B	C28B	40.64(14)
C29A	Fe1A	C25A	40.90(13)	C27B	Fe1B	C29B	68.54(14)
C29A	Fe1A	C26A	68.62(13)	C28B	Fe1B	C20B	154.06(14)
C29A	Fe1A	C27A	68.63(14)	C29B	Fe1B	C20B	119.18(13)
C29A	Fe1A	C28A	40.58(14)	C29B	Fe1B	C28B	40.57(14)
C4A	N1A	C14A	118.7(3)	C4B	N1B	C14B	117.6(3)
C4A	N1A	Re1A	126.6(2)	C4B	N1B	Re1B	127.3(2)
C14A	N1A	Re1A	114.60(19)	C14B	N1B	Re1B	114.9(2)
C13A	N2A	C15A	118.0(3)	C13B	N2B	C15B	117.8(3)
C13A	N2A	Re1A	126.9(2)	C13B	N2B	Re1B	127.6(2)
C15A	N2A	Re1A	115.11(18)	C15B	N2B	Re1B	114.5(2)
C16A	O4A	Re1A	125.8(2)	C16B	O4B	Re1B	127.2(2)
C1A	Re1A	C2A	89.37(14)	C1B	Re1B	C2B	90.40(17)
C1A	Re1A	C3A	88.52(14)	C1B	Re1B	C3B	87.55(16)
C1A	Re1A	N1A	97.05(12)	C1B	Re1B	N1B	93.49(14)
C1A	Re1A	N2A	171.83(12)	C1B	Re1B	N2B	169.13(14)
C1A	Re1A	O4A	96.39(12)	C1B	Re1B	O4B	98.50(13)
C2A	Re1A	N1A	173.58(12)	C2B	Re1B	C3B	86.15(14)
C2A	Re1A	N2A	97.76(12)	C2B	Re1B	N1B	174.47(14)
C2A	Re1A	O4A	96.84(11)	C2B	Re1B	N2B	99.96(14)
C3A	Re1A	C2A	86.00(14)	C2B	Re1B	O4B	95.81(13)
C3A	Re1A	N1A	94.31(12)	C3B	Re1B	N1B	97.92(12)
C3A	Re1A	N2A	95.92(12)	C3B	Re1B	N2B	96.34(13)
C3A	Re1A	O4A	174.35(12)	C3B	Re1B	O4B	173.61(12)
N2A	Re1A	N1A	75.83(9)	N2B	Re1B	N1B	75.95(10)
O4A	Re1A	N1A	82.33(9)	O4B	Re1B	N1B	79.74(10)
O4A	Re1A	N2A	78.88(9)	O4B	Re1B	N2B	77.34(9)

Table S10 Torsion angles for **3**.

A	B	C	D	Angle/°	A	B	C	D	Angle/°
C4A	C5A	C6A	C7A	1.3(5)	C4B	C5B	C6B	C7B	0.0(5)
C4A	N1A	Re1A	C1A	1.2(3)	C4B	N1B	Re1B	C1B	0.2(3)
C4A	N1A	Re1A	C2A	180(16)	C4B	N1B	Re1B	C2B	134.9(13)
C4A	N1A	Re1A	C3A	-87.8(3)	C4B	N1B	Re1B	C3B	-87.8(3)
C4A	N1A	Re1A	N2A	177.2(3)	C4B	N1B	Re1B	N2B	177.6(3)
C4A	N1A	Re1A	O4A	96.8(3)	C4B	N1B	Re1B	O4B	98.2(3)
C5A	C4A	N1A	C14A	-0.3(5)	C5B	C4B	N1B	C14B	0.0(5)
C5A	C4A	N1A	Re1A	178.3(2)	C5B	C4B	N1B	Re1B	-175.1(2)
C5A	C6A	C7A	C8A	179.2(3)	C5B	C6B	C7B	C8B	-178.8(3)
C5A	C6A	C7A	C14A	-0.4(5)	C5B	C6B	C7B	C14B	0.7(5)
C6A	C7A	C8A	C9A	-177.8(3)	C6B	C7B	C8B	C9B	-178.3(4)
C6A	C7A	C14A	C15A	177.4(3)	C6B	C7B	C14B	C15B	177.8(3)
C6A	C7A	C14A	N1A	-0.8(4)	C6B	C7B	C14B	N1B	-1.0(5)
C7A	C8A	C9A	C10A	1.0(5)	C7B	C8B	C9B	C10B	0.0(6)
C7A	C14A	C15A	C10A	-0.1(4)	C7B	C14B	C15B	C10B	1.0(5)
C7A	C14A	C15A	N2A	-179.2(3)	C7B	C14B	C15B	N2B	-178.2(3)
C7A	C14A	N1A	C4A	1.2(4)	C7B	C14B	N1B	C4B	0.7(5)
C7A	C14A	N1A	Re1A	-177.6(2)	C7B	C14B	N1B	Re1B	176.4(2)
C8A	C7A	C14A	C15A	-2.2(4)	C8B	C7B	C14B	C15B	-2.6(5)
C8A	C7A	C14A	N1A	179.6(3)	C8B	C7B	C14B	N1B	178.5(3)
C8A	C9A	C10A	C11A	176.6(3)	C8B	C9B	C10B	C11B	177.7(4)
C8A	C9A	C10A	C15A	-3.3(5)	C8B	C9B	C10B	C15B	-1.7(5)
C9A	C10A	C11A	C12A	-179.1(3)	C9B	C10B	C11B	C12B	-178.9(4)
C9A	C10A	C15A	C14A	2.8(4)	C9B	C10B	C15B	C14B	1.1(5)
C9A	C10A	C15A	N2A	-178.2(3)	C9B	C10B	C15B	N2B	-179.6(3)
C10A	C11A	C12A	C13A	-1.9(5)	C10B	C11B	C12B	C13B	-1.8(5)
C10A	C15A	N2A	C13A	-3.4(4)	C10B	C15B	N2B	C13B	-1.0(5)
C10A	C15A	N2A	Re1A	178.1(2)	C10B	C15B	N2B	Re1B	-177.8(3)
C11A	C10A	C15A	C14A	-177.1(3)	C11B	C10B	C15B	C14B	-178.3(3)
C11A	C10A	C15A	N2A	1.9(4)	C11B	C10B	C15B	N2B	1.0(5)
C11A	C12A	C13A	N2A	0.4(5)	C11B	C12B	C13B	N2B	1.8(5)
C12A	C13A	N2A	C15A	2.2(4)	C12B	C13B	N2B	C15B	-0.4(5)
C12A	C13A	N2A	Re1A	-179.5(2)	C12B	C13B	N2B	Re1B	175.9(3)
C13A	N2A	Re1A	C1A	-144.9(8)	C13B	N2B	Re1B	C1B	-164.5(7)
C13A	N2A	Re1A	C2A	5.7(3)	C13B	N2B	Re1B	C2B	-2.3(3)
C13A	N2A	Re1A	C3A	92.4(3)	C13B	N2B	Re1B	C3B	84.9(3)
C13A	N2A	Re1A	N1A	-174.6(3)	C13B	N2B	Re1B	N1B	-178.5(3)
C13A	N2A	Re1A	O4A	-89.8(3)	C13B	N2B	Re1B	O4B	-96.0(3)
C14A	C7A	C8A	C9A	1.8(5)	C14B	C7B	C8B	C9B	2.1(5)
C14A	C15A	N2A	C13A	175.6(3)	C14B	C15B	N2B	C13B	178.3(3)

C14A	C15A	N2A	Re1A	-2.9(3)	C14B	C15B	N2B	Re1B	1.5(4)
C14A	N1A	Re1A	C1A	179.9(2)	C14B	N1B	Re1B	C1B	-175.0(2)
C14A	N1A	Re1A	C2A	-1.6(12)	C14B	N1B	Re1B	C2B	-40.3(15)
C14A	N1A	Re1A	C3A	90.9(2)	C14B	N1B	Re1B	C3B	97.0(2)
C14A	N1A	Re1A	N2A	-4.11(19)	C14B	N1B	Re1B	N2B	2.4(2)
C14A	N1A	Re1A	O4A	-84.5(2)	C14B	N1B	Re1B	O4B	-77.0(2)
C15A	C10A	C11A	C12A	0.9(5)	C15B	C10B	C11B	C12B	0.5(5)
C15A	C14A	N1A	C4A	-177.1(3)	C15B	C14B	N1B	C4B	-178.2(3)
C15A	C14A	N1A	Re1A	4.1(3)	C15B	C14B	N1B	Re1B	-2.5(4)
C15A	N2A	Re1A	C1A	33.4(9)	C15B	N2B	Re1B	C1B	11.9(8)
C15A	N2A	Re1A	C2A	-176.0(2)	C15B	N2B	Re1B	C2B	174.2(2)
C15A	N2A	Re1A	C3A	-89.3(2)	C15B	N2B	Re1B	C3B	-98.7(2)
C15A	N2A	Re1A	N1A	3.71(19)	C15B	N2B	Re1B	N1B	-2.0(2)
C15A	N2A	Re1A	O4A	88.5(2)	C15B	N2B	Re1B	O4B	80.4(2)
C16A	C17A	C18A	C19A	-61.0(4)	C16B	C17B	C18B	C19B	-64.5(4)
C16A	O4A	Re1A	C1A	35.3(3)	C16B	O4B	Re1B	C1B	-21.1(3)
C16A	O4A	Re1A	C2A	125.5(2)	C16B	O4B	Re1B	C2B	70.2(3)
C16A	O4A	Re1A	C3A	-114.7(11)	C16B	O4B	Re1B	C3B	178(8)
C16A	O4A	Re1A	N1A	-61.0(2)	C16B	O4B	Re1B	N1B	-113.1(3)
C16A	O4A	Re1A	N2A	-137.9(2)	C16B	O4B	Re1B	N2B	169.1(3)
C17A	C16A	O4A	Re1A	170.65(19)	C17B	C16B	O4B	Re1B	176.4(2)
C17A	C18A	C19A	C20A	176.5(3)	C17B	C18B	C19B	C20B	177.5(3)
C18A	C19A	C20A	C21A	-21.2(5)	C18B	C19B	C20B	C21B	-17.5(5)
C18A	C19A	C20A	C24A	161.4(3)	C18B	C19B	C20B	C24B	165.7(3)
C18A	C19A	C20A	Fe1A	72.3(4)	C18B	C19B	C20B	Fe1B	75.2(4)
C19A	C20A	C21A	C22A	-177.9(3)	C19B	C20B	C21B	C22B	-177.0(3)
C19A	C20A	C21A	Fe1A	123.0(3)	C19B	C20B	C21B	Fe1B	123.6(4)
C19A	C20A	C24A	C23A	177.9(3)	C19B	C20B	C24B	C23B	177.4(3)
C19A	C20A	C24A	Fe1A	-123.2(3)	C19B	C20B	C24B	Fe1B	-123.7(3)
C19A	C20A	Fe1A	C21A	-124.5(4)	C19B	C20B	Fe1B	C21B	-122.8(4)
C19A	C20A	Fe1A	C22A	-162.2(3)	C19B	C20B	Fe1B	C22B	-160.1(3)
C19A	C20A	Fe1A	C23A	154.2(3)	C19B	C20B	Fe1B	C23B	155.7(3)
C19A	C20A	Fe1A	C24A	116.6(4)	C19B	C20B	Fe1B	C24B	118.9(4)
C19A	C20A	Fe1A	C25A	-8.0(3)	C19B	C20B	Fe1B	C25B	-9.3(3)
C19A	C20A	Fe1A	C26A	-49.0(3)	C19B	C20B	Fe1B	C26B	-50.0(3)
C19A	C20A	Fe1A	C27A	-79.6(5)	C19B	C20B	Fe1B	C27B	-80.8(6)
C19A	C20A	Fe1A	C28A	66.3(5)	C19B	C20B	Fe1B	C28B	66.3(4)
C19A	C20A	Fe1A	C29A	34.5(3)	C19B	C20B	Fe1B	C29B	33.5(3)
C20A	C21A	C22A	C23A	0.3(4)	C20B	C21B	C22B	C23B	-0.4(4)
C20A	C21A	C22A	Fe1A	-59.6(2)	C20B	C21B	C22B	Fe1B	-59.6(2)
C20A	C21A	Fe1A	C22A	119.8(3)	C20B	C21B	Fe1B	C22B	119.9(3)
C20A	C21A	Fe1A	C23A	82.0(2)	C20B	C21B	Fe1B	C23B	81.8(2)
C20A	C21A	Fe1A	C24A	37.78(19)	C20B	C21B	Fe1B	C24B	38.18(18)
C20A	C21A	Fe1A	C25A	-80.8(2)	C20B	C21B	Fe1B	C25B	-83.7(2)
C20A	C21A	Fe1A	C26A	-123.3(2)	C20B	C21B	Fe1B	C26B	-126.3(2)
C20A	C21A	Fe1A	C27A	-164.43(19)	C20B	C21B	Fe1B	C27B	-166.51(19)
C20A	C21A	Fe1A	C28A	164.4(4)	C20B	C21B	Fe1B	C28B	165.3(5)
C20A	C21A	Fe1A	C29A	-46.9(4)	C20B	C21B	Fe1B	C29B	-50.9(4)

C20A	C24A	Fe1A	C21A	-37.88(19)	C20B	C24B	Fe1B	C21B	-38.29(19)
C20A	C24A	Fe1A	C22A	-82.3(2)	C20B	C24B	Fe1B	C22B	-82.3(2)
C20A	C24A	Fe1A	C23A	-119.7(3)	C20B	C24B	Fe1B	C23B	-120.5(3)
C20A	C24A	Fe1A	C25A	73.9(2)	C20B	C24B	Fe1B	C25B	71.1(2)
C20A	C24A	Fe1A	C26A	40.8(5)	C20B	C24B	Fe1B	C26B	35.6(6)
C20A	C24A	Fe1A	C27A	-167.6(3)	C20B	C24B	Fe1B	C27B	-167.4(3)
C20A	C24A	Fe1A	C28A	158.00(19)	C20B	C24B	Fe1B	C28B	156.2(2)
C20A	C24A	Fe1A	C29A	115.7(2)	C20B	C24B	Fe1B	C29B	113.1(2)
C21A	C20A	C24A	C23A	0.0(4)	C21B	C20B	C24B	C23B	0.0(4)
C21A	C20A	C24A	Fe1A	59.0(2)	C21B	C20B	C24B	Fe1B	58.9(2)
C21A	C20A	Fe1A	C22A	-37.7(2)	C21B	C20B	Fe1B	C22B	-37.37(19)
C21A	C20A	Fe1A	C23A	-81.3(2)	C21B	C20B	Fe1B	C23B	-81.5(2)
C21A	C20A	Fe1A	C24A	-118.9(3)	C21B	C20B	Fe1B	C24B	-118.4(3)
C21A	C20A	Fe1A	C25A	116.6(2)	C21B	C20B	Fe1B	C25B	113.5(2)
C21A	C20A	Fe1A	C26A	75.6(2)	C21B	C20B	Fe1B	C26B	72.7(2)
C21A	C20A	Fe1A	C27A	45.0(5)	C21B	C20B	Fe1B	C27B	41.9(6)
C21A	C20A	Fe1A	C28A	-169.1(3)	C21B	C20B	Fe1B	C28B	-170.9(3)
C21A	C20A	Fe1A	C29A	158.99(19)	C21B	C20B	Fe1B	C29B	156.28(19)
C21A	C22A	C23A	C24A	-0.3(4)	C21B	C22B	C23B	C24B	0.4(4)
C21A	C22A	C23A	Fe1A	-59.5(2)	C21B	C22B	C23B	Fe1B	-59.1(2)
C21A	C22A	Fe1A	C20A	37.21(19)	C21B	C22B	Fe1B	C20B	37.4(2)
C21A	C22A	Fe1A	C23A	118.6(3)	C21B	C22B	Fe1B	C23B	118.9(3)
C21A	C22A	Fe1A	C24A	80.8(2)	C21B	C22B	Fe1B	C24B	81.3(2)
C21A	C22A	Fe1A	C25A	-46.8(4)	C21B	C22B	Fe1B	C25B	-49.1(4)
C21A	C22A	Fe1A	C26A	-81.4(2)	C21B	C22B	Fe1B	C26B	-83.2(2)
C21A	C22A	Fe1A	C27A	-124.0(2)	C21B	C22B	Fe1B	C27B	-125.7(2)
C21A	C22A	Fe1A	C28A	-165.4(2)	C21B	C22B	Fe1B	C28B	-166.0(2)
C21A	C22A	Fe1A	C29A	160.9(4)	C21B	C22B	Fe1B	C29B	163.5(5)
C22A	C21A	Fe1A	C20A	-119.8(3)	C22B	C21B	Fe1B	C20B	-119.9(3)
C22A	C21A	Fe1A	C23A	-37.9(2)	C22B	C21B	Fe1B	C23B	-38.1(2)
C22A	C21A	Fe1A	C24A	-82.1(2)	C22B	C21B	Fe1B	C24B	-81.7(2)
C22A	C21A	Fe1A	C25A	159.4(2)	C22B	C21B	Fe1B	C25B	156.4(2)
C22A	C21A	Fe1A	C26A	116.8(2)	C22B	C21B	Fe1B	C26B	113.8(2)
C22A	C21A	Fe1A	C27A	75.7(2)	C22B	C21B	Fe1B	C27B	73.6(3)
C22A	C21A	Fe1A	C28A	44.6(5)	C22B	C21B	Fe1B	C28B	45.3(6)
C22A	C21A	Fe1A	C29A	-166.7(3)	C22B	C21B	Fe1B	C29B	-170.8(3)
C22A	C23A	C24A	C20A	0.2(4)	C22B	C23B	C24B	C20B	-0.2(4)
C22A	C23A	C24A	Fe1A	-59.0(2)	C22B	C23B	C24B	Fe1B	-59.4(2)
C22A	C23A	Fe1A	C20A	82.2(2)	C22B	C23B	Fe1B	C20B	81.9(2)
C22A	C23A	Fe1A	C21A	38.4(2)	C22B	C23B	Fe1B	C21B	37.9(2)
C22A	C23A	Fe1A	C24A	119.4(3)	C22B	C23B	Fe1B	C24B	119.0(3)
C22A	C23A	Fe1A	C25A	159.4(4)	C22B	C23B	Fe1B	C25B	157.5(5)
C22A	C23A	Fe1A	C26A	-45.8(4)	C22B	C23B	Fe1B	C26B	-47.1(4)
C22A	C23A	Fe1A	C27A	-80.9(2)	C22B	C23B	Fe1B	C27B	-82.4(2)
C22A	C23A	Fe1A	C28A	-123.9(2)	C22B	C23B	Fe1B	C28B	-125.6(2)
C22A	C23A	Fe1A	C29A	-165.7(2)	C22B	C23B	Fe1B	C29B	-166.9(2)
C23A	C22A	Fe1A	C20A	-81.4(2)	C23B	C22B	Fe1B	C20B	-81.5(2)
C23A	C22A	Fe1A	C21A	-118.6(3)	C23B	C22B	Fe1B	C21B	-118.9(3)

C23A	C22A	Fe1A	C24A	-37.8(2)	C23B	C22B	Fe1B	C24B	-37.57(19)
C23A	C22A	Fe1A	C25A	-165.4(3)	C23B	C22B	Fe1B	C25B	-168.0(3)
C23A	C22A	Fe1A	C26A	160.1(2)	C23B	C22B	Fe1B	C26B	158.0(2)
C23A	C22A	Fe1A	C27A	117.4(2)	C23B	C22B	Fe1B	C27B	115.4(2)
C23A	C22A	Fe1A	C28A	76.0(2)	C23B	C22B	Fe1B	C28B	75.1(2)
C23A	C22A	Fe1A	C29A	42.4(6)	C23B	C22B	Fe1B	C29B	44.7(6)
C23A	C24A	Fe1A	C20A	119.7(3)	C23B	C24B	Fe1B	C20B	120.5(3)
C23A	C24A	Fe1A	C21A	81.8(2)	C23B	C24B	Fe1B	C21B	82.2(2)
C23A	C24A	Fe1A	C22A	37.5(2)	C23B	C24B	Fe1B	C22B	38.1(2)
C23A	C24A	Fe1A	C25A	-166.38(19)	C23B	C24B	Fe1B	C25B	-168.5(2)
C23A	C24A	Fe1A	C26A	160.5(4)	C23B	C24B	Fe1B	C26B	156.0(5)
C23A	C24A	Fe1A	C27A	-47.9(4)	C23B	C24B	Fe1B	C27B	-46.9(4)
C23A	C24A	Fe1A	C28A	-82.3(2)	C23B	C24B	Fe1B	C28B	-83.3(2)
C23A	C24A	Fe1A	C29A	-124.6(2)	C23B	C24B	Fe1B	C29B	-126.4(2)
C24A	C20A	C21A	C22A	-0.2(4)	C24B	C20B	C21B	C22B	0.3(4)
C24A	C20A	C21A	Fe1A	-59.4(2)	C24B	C20B	C21B	Fe1B	-59.1(2)
C24A	C20A	Fe1A	C21A	118.9(3)	C24B	C20B	Fe1B	C21B	118.4(3)
C24A	C20A	Fe1A	C22A	81.2(2)	C24B	C20B	Fe1B	C22B	81.0(2)
C24A	C20A	Fe1A	C23A	37.62(19)	C24B	C20B	Fe1B	C23B	36.85(19)
C24A	C20A	Fe1A	C25A	-124.5(2)	C24B	C20B	Fe1B	C25B	-128.2(2)
C24A	C20A	Fe1A	C26A	-165.52(19)	C24B	C20B	Fe1B	C26B	-168.9(2)
C24A	C20A	Fe1A	C27A	163.9(4)	C24B	C20B	Fe1B	C27B	160.3(5)
C24A	C20A	Fe1A	C28A	-50.3(4)	C24B	C20B	Fe1B	C28B	-52.6(4)
C24A	C20A	Fe1A	C29A	-82.1(2)	C24B	C20B	Fe1B	C29B	-85.3(2)
C24A	C23A	Fe1A	C20A	-37.25(18)	C24B	C23B	Fe1B	C20B	-37.13(19)
C24A	C23A	Fe1A	C21A	-81.0(2)	C24B	C23B	Fe1B	C21B	-81.1(2)
C24A	C23A	Fe1A	C22A	-119.4(3)	C24B	C23B	Fe1B	C22B	-119.0(3)
C24A	C23A	Fe1A	C25A	40.0(5)	C24B	C23B	Fe1B	C25B	38.5(6)
C24A	C23A	Fe1A	C26A	-165.2(3)	C24B	C23B	Fe1B	C26B	-166.1(3)
C24A	C23A	Fe1A	C27A	159.69(19)	C24B	C23B	Fe1B	C27B	158.6(2)
C24A	C23A	Fe1A	C28A	116.7(2)	C24B	C23B	Fe1B	C28B	115.4(2)
C24A	C23A	Fe1A	C29A	74.9(2)	C24B	C23B	Fe1B	C29B	74.0(2)
C25A	C26A	C27A	C28A	-0.6(4)	C25B	C26B	C27B	C28B	-0.8(4)
C25A	C26A	C27A	Fe1A	59.0(2)	C25B	C26B	C27B	Fe1B	58.8(2)
C25A	C26A	Fe1A	C20A	74.4(2)	C25B	C26B	Fe1B	C20B	73.0(2)
C25A	C26A	Fe1A	C21A	115.7(2)	C25B	C26B	Fe1B	C21B	113.7(2)
C25A	C26A	Fe1A	C22A	158.6(2)	C25B	C26B	Fe1B	C22B	156.4(2)
C25A	C26A	Fe1A	C23A	-168.7(3)	C25B	C26B	Fe1B	C23B	-170.6(3)
C25A	C26A	Fe1A	C24A	43.5(5)	C25B	C26B	Fe1B	C24B	45.0(6)
C25A	C26A	Fe1A	C27A	-119.5(3)	C25B	C26B	Fe1B	C27B	-119.9(3)
C25A	C26A	Fe1A	C28A	-81.6(2)	C25B	C26B	Fe1B	C28B	-82.0(2)
C25A	C26A	Fe1A	C29A	-37.9(2)	C25B	C26B	Fe1B	C29B	-38.2(2)
C25A	C29A	Fe1A	C20A	-80.8(2)	C25B	C29B	Fe1B	C20B	-82.3(2)
C25A	C29A	Fe1A	C21A	-47.6(4)	C25B	C29B	Fe1B	C21B	-46.7(4)
C25A	C29A	Fe1A	C22A	162.7(4)	C25B	C29B	Fe1B	C22B	157.4(5)
C25A	C29A	Fe1A	C23A	-164.8(2)	C25B	C29B	Fe1B	C23B	-166.94(19)
C25A	C29A	Fe1A	C24A	-123.2(2)	C25B	C29B	Fe1B	C24B	-125.72(19)
C25A	C29A	Fe1A	C26A	37.7(2)	C25B	C29B	Fe1B	C26B	37.99(19)

C25A	C29A	Fe1A	C27A	81.5(2)	C25B	C29B	Fe1B	C27B	81.7(2)
C25A	C29A	Fe1A	C28A	118.9(3)	C25B	C29B	Fe1B	C28B	119.1(3)
C26A	C25A	C29A	C28A	0.4(4)	C26B	C25B	C29B	C28B	-0.5(4)
C26A	C25A	C29A	Fe1A	-59.4(2)	C26B	C25B	C29B	Fe1B	-60.0(2)
C26A	C25A	Fe1A	C20A	-124.1(2)	C26B	C25B	Fe1B	C20B	-126.0(2)
C26A	C25A	Fe1A	C21A	-82.0(2)	C26B	C25B	Fe1B	C21B	-83.2(2)
C26A	C25A	Fe1A	C22A	-48.5(4)	C26B	C25B	Fe1B	C22B	-49.2(4)
C26A	C25A	Fe1A	C23A	164.5(4)	C26B	C25B	Fe1B	C23B	163.2(5)
C26A	C25A	Fe1A	C24A	-164.71(19)	C26B	C25B	Fe1B	C24B	-166.2(2)
C26A	C25A	Fe1A	C27A	37.5(2)	C26B	C25B	Fe1B	C27B	37.2(2)
C26A	C25A	Fe1A	C28A	81.4(2)	C26B	C25B	Fe1B	C28B	81.0(2)
C26A	C25A	Fe1A	C29A	119.2(3)	C26B	C25B	Fe1B	C29B	118.7(3)
C26A	C27A	C28A	C29A	0.9(4)	C26B	C27B	C28B	C29B	0.5(4)
C26A	C27A	C28A	Fe1A	59.6(2)	C26B	C27B	C28B	Fe1B	59.3(3)
C26A	C27A	Fe1A	C20A	40.1(5)	C26B	C27B	Fe1B	C20B	39.6(6)
C26A	C27A	Fe1A	C21A	74.4(2)	C26B	C27B	Fe1B	C21B	72.3(3)
C26A	C27A	Fe1A	C22A	116.4(2)	C26B	C27B	Fe1B	C22B	113.3(2)
C26A	C27A	Fe1A	C23A	158.7(2)	C26B	C27B	Fe1B	C23B	156.3(2)
C26A	C27A	Fe1A	C24A	-166.7(3)	C26B	C27B	Fe1B	C24B	-170.8(3)
C26A	C27A	Fe1A	C25A	-37.6(2)	C26B	C27B	Fe1B	C25B	-37.5(2)
C26A	C27A	Fe1A	C28A	-119.0(3)	C26B	C27B	Fe1B	C28B	-119.0(3)
C26A	C27A	Fe1A	C29A	-81.7(2)	C26B	C27B	Fe1B	C29B	-81.7(2)
C27A	C26A	Fe1A	C20A	-166.02(19)	C27B	C26B	Fe1B	C20B	-167.1(2)
C27A	C26A	Fe1A	C21A	-124.7(2)	C27B	C26B	Fe1B	C21B	-126.4(2)
C27A	C26A	Fe1A	C22A	-81.8(2)	C27B	C26B	Fe1B	C22B	-83.8(2)
C27A	C26A	Fe1A	C23A	-49.1(4)	C27B	C26B	Fe1B	C23B	-50.8(4)
C27A	C26A	Fe1A	C24A	163.0(4)	C27B	C26B	Fe1B	C24B	164.9(5)
C27A	C26A	Fe1A	C25A	119.5(3)	C27B	C26B	Fe1B	C25B	119.9(3)
C27A	C26A	Fe1A	C28A	37.9(2)	C27B	C26B	Fe1B	C28B	37.8(2)
C27A	C26A	Fe1A	C29A	81.7(2)	C27B	C26B	Fe1B	C29B	81.6(2)
C27A	C28A	C29A	C25A	-0.8(4)	C27B	C28B	C29B	C25B	0.0(4)
C27A	C28A	C29A	Fe1A	58.9(2)	C27B	C28B	C29B	Fe1B	58.8(3)
C27A	C28A	Fe1A	C20A	-164.6(3)	C27B	C28B	Fe1B	C20B	-166.4(3)
C27A	C28A	Fe1A	C21A	40.3(5)	C27B	C28B	Fe1B	C21B	36.0(6)
C27A	C28A	Fe1A	C22A	74.9(2)	C27B	C28B	Fe1B	C22B	71.7(3)
C27A	C28A	Fe1A	C23A	116.6(2)	C27B	C28B	Fe1B	C23B	113.8(2)
C27A	C28A	Fe1A	C24A	159.7(2)	C27B	C28B	Fe1B	C24B	156.6(2)
C27A	C28A	Fe1A	C25A	-81.8(2)	C27B	C28B	Fe1B	C25B	-81.7(2)
C27A	C28A	Fe1A	C26A	-37.8(2)	C27B	C28B	Fe1B	C26B	-37.7(2)
C27A	C28A	Fe1A	C29A	-119.8(3)	C27B	C28B	Fe1B	C29B	-119.8(3)
C28A	C27A	Fe1A	C20A	159.0(4)	C28B	C27B	Fe1B	C20B	158.6(4)
C28A	C27A	Fe1A	C21A	-166.6(2)	C28B	C27B	Fe1B	C21B	-168.7(2)
C28A	C27A	Fe1A	C22A	-124.6(2)	C28B	C27B	Fe1B	C22B	-127.7(2)
C28A	C27A	Fe1A	C23A	-82.3(2)	C28B	C27B	Fe1B	C23B	-84.7(2)
C28A	C27A	Fe1A	C24A	-47.7(4)	C28B	C27B	Fe1B	C24B	-51.8(4)
C28A	C27A	Fe1A	C25A	81.4(2)	C28B	C27B	Fe1B	C25B	81.6(2)
C28A	C27A	Fe1A	C26A	119.0(3)	C28B	C27B	Fe1B	C26B	119.0(3)
C28A	C27A	Fe1A	C29A	37.3(2)	C28B	C27B	Fe1B	C29B	37.3(2)

C28A	C29A	Fe1A	C20A	160.25(19)	C28B	C29B	Fe1B	C20B	158.65(18)
C28A	C29A	Fe1A	C21A	-166.5(3)	C28B	C29B	Fe1B	C21B	-165.8(3)
C28A	C29A	Fe1A	C22A	43.8(5)	C28B	C29B	Fe1B	C22B	38.4(6)
C28A	C29A	Fe1A	C23A	76.3(2)	C28B	C29B	Fe1B	C23B	74.0(2)
C28A	C29A	Fe1A	C24A	117.8(2)	C28B	C29B	Fe1B	C24B	115.22(19)
C28A	C29A	Fe1A	C25A	-118.9(3)	C28B	C29B	Fe1B	C25B	-119.1(3)
C28A	C29A	Fe1A	C26A	-81.2(2)	C28B	C29B	Fe1B	C26B	-81.1(2)
C28A	C29A	Fe1A	C27A	-37.41(19)	C28B	C29B	Fe1B	C27B	-37.40(19)
C29A	C25A	C26A	C27A	0.2(4)	C29B	C25B	C26B	C27B	0.8(4)
C29A	C25A	C26A	Fe1A	59.2(2)	C29B	C25B	C26B	Fe1B	60.1(2)
C29A	C25A	Fe1A	C20A	116.7(2)	C29B	C25B	Fe1B	C20B	115.3(2)
C29A	C25A	Fe1A	C21A	158.8(2)	C29B	C25B	Fe1B	C21B	158.09(19)
C29A	C25A	Fe1A	C22A	-167.7(3)	C29B	C25B	Fe1B	C22B	-167.9(3)
C29A	C25A	Fe1A	C23A	45.3(5)	C29B	C25B	Fe1B	C23B	44.6(6)
C29A	C25A	Fe1A	C24A	76.1(2)	C29B	C25B	Fe1B	C24B	75.1(2)
C29A	C25A	Fe1A	C26A	-119.2(3)	C29B	C25B	Fe1B	C26B	-118.7(3)
C29A	C25A	Fe1A	C27A	-81.7(2)	C29B	C25B	Fe1B	C27B	-81.5(2)
C29A	C25A	Fe1A	C28A	-37.8(2)	C29B	C25B	Fe1B	C28B	-37.65(19)
C29A	C28A	Fe1A	C20A	-44.7(4)	C29B	C28B	Fe1B	C20B	-46.6(4)
C29A	C28A	Fe1A	C21A	160.1(4)	C29B	C28B	Fe1B	C21B	155.8(5)
C29A	C28A	Fe1A	C22A	-165.2(2)	C29B	C28B	Fe1B	C22B	-168.53(19)
C29A	C28A	Fe1A	C23A	-123.6(2)	C29B	C28B	Fe1B	C23B	-126.46(19)
C29A	C28A	Fe1A	C24A	-80.4(2)	C29B	C28B	Fe1B	C24B	-83.6(2)
C29A	C28A	Fe1A	C25A	38.05(19)	C29B	C28B	Fe1B	C25B	38.05(19)
C29A	C28A	Fe1A	C26A	82.0(2)	C29B	C28B	Fe1B	C26B	82.1(2)
C29A	C28A	Fe1A	C27A	119.8(3)	C29B	C28B	Fe1B	C27B	119.8(3)
Fe1A	C20A	C21A	C22A	59.1(2)	Fe1B	C20B	C21B	C22B	59.4(3)
Fe1A	C20A	C24A	C23A	-59.0(2)	Fe1B	C20B	C24B	C23B	-58.9(2)
Fe1A	C21A	C22A	C23A	60.0(2)	Fe1B	C21B	C22B	C23B	59.2(2)
Fe1A	C22A	C23A	C24A	59.2(2)	Fe1B	C22B	C23B	C24B	59.5(2)
Fe1A	C23A	C24A	C20A	59.2(2)	Fe1B	C23B	C24B	C20B	59.1(2)
Fe1A	C25A	C26A	C27A	-59.0(2)	Fe1B	C25B	C26B	C27B	-59.3(3)
Fe1A	C25A	C29A	C28A	59.8(2)	Fe1B	C25B	C29B	C28B	59.4(2)
Fe1A	C26A	C27A	C28A	-59.6(2)	Fe1B	C26B	C27B	C28B	-59.6(3)
Fe1A	C27A	C28A	C29A	-58.8(2)	Fe1B	C27B	C28B	C29B	-58.9(2)
Fe1A	C28A	C29A	C25A	-59.6(2)	Fe1B	C28B	C29B	C25B	-58.8(2)
N1A	C4A	C5A	C6A	-0.9(5)	N1B	C4B	C5B	C6B	-0.3(5)
N1A	C14A	C15A	C10A	178.2(3)	N1B	C14B	C15B	C10B	180.0(3)
N1A	C14A	C15A	N2A	-0.8(4)	N1B	C14B	C15B	N2B	0.7(4)
O1A	C1A	Re1A	C2A	56(20)	O1B	C1B	Re1B	C2B	156(3)
O1A	C1A	Re1A	C3A	-30(20)	O1B	C1B	Re1B	C3B	70(3)
O1A	C1A	Re1A	N1A	-124(20)	O1B	C1B	Re1B	N1B	-28(3)
O1A	C1A	Re1A	N2A	-153(19)	O1B	C1B	Re1B	N2B	-41(4)
O1A	C1A	Re1A	O4A	153(20)	O1B	C1B	Re1B	O4B	-108(3)
O2A	C2A	Re1A	C1A	-92(4)	O2B	C2B	Re1B	C1B	-67(12)
O2A	C2A	Re1A	C3A	-4(4)	O2B	C2B	Re1B	C3B	21(12)
O2A	C2A	Re1A	N1A	89(4)	O2B	C2B	Re1B	N1B	158(11)
O2A	C2A	Re1A	N2A	92(4)	O2B	C2B	Re1B	N2B	117(12)

O2A	C2A	Re1A	O4A	171(4)	O2B	C2B	Re1B	O4B	-165(12)
O3A	C3A	Re1A	C1A	25(8)	O3B	C3B	Re1B	C1B	57(5)
O3A	C3A	Re1A	C2A	-64(8)	O3B	C3B	Re1B	C2B	-34(5)
O3A	C3A	Re1A	N1A	122(8)	O3B	C3B	Re1B	N1B	150(5)
O3A	C3A	Re1A	N2A	-161(8)	O3B	C3B	Re1B	N2B	-133(5)
O3A	C3A	Re1A	O4A	176(100)	O3B	C3B	Re1B	O4B	-142(4)
O4A	C16A	C17A	C18A	-55.6(4)	O4B	C16B	C17B	C18B	-27.8(4)
O5A	C16A	C17A	C18A	123.4(3)	O5B	C16B	C17B	C18B	152.9(3)
O5A	C16A	O4A	Re1A	-8.3(5)	O5B	C16B	O4B	Re1B	-4.4(4)
